# Synthesis, α-Glucosidase, α-Amylase, and Aldol Reductase Inhibitory Activity with Molecular Docking Study of Novel Imidazo[1,2-*a*]pyridine Derivatives

**DOI:** 10.1021/acsomega.4c05619

**Published:** 2024-10-11

**Authors:** Betül Kaya, Ulviye Acar Çevik, Bilge Çiftçi, Hatice Esra Duran, Cüneyt Türkeş, Mesut Işık, Hayrani Eren Bostancı, Zafer Asım Kaplancıklı, Şükrü Beydemir

**Affiliations:** † Department of Pharmaceutical Chemistry, Faculty of Pharmacy, Zonguldak Bulent Ecevit University, 67600 Zonguldak, Turkey; ‡ Department of Pharmaceutical Chemistry, Faculty of Pharmacy, Anadolu University, 26470 Eskişehir, Turkey; § Vocational School of Health Services, Bilecik Şeyh Edebali University, 11230 Bilecik, Turkey; ∥ Department of Medical Biochemistry, Faculty of Medicine, Kafkas University, 36100 Kars, Turkey; ⊥ Department of Biochemistry, Faculty of Pharmacy, Erzincan Binali Yıldırım University, 24002 Erzincan, Turkey; # Department of Bioengineering, Faculty of Engineering, Bilecik Şeyh Edebali University, 11230 Bilecik, Turkey; ¶ Department of Biochemistry, Faculty of Pharmacy, Sivas Cumhuriyet University, 58140 Sivas, Turkey; ∇ Department of Biochemistry, Faculty of Pharmacy, Anadolu University, 26470 Eskişehir, Turkey

## Abstract

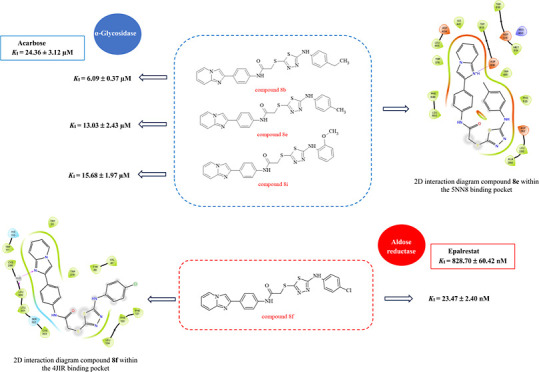

Inhibition ofaldose reductase (AR), α-glycosidase (α-GLY), and α-amylase (α-AMY) are some of the essential targets in diabetes mellitus (DM). Here, a series of imidazo[1,2-*a*]pyridine-based 1,3,4-thiadiazole derivatives (**8a**–**k**) were successfully synthesized and characterized using ^1^H NMR, ^13^C NMR, and HRMS spectroscopic techniques. The inhibition effects of the synthesized derivatives against AR, α-GLY, and α-AMY were evaluated using both in vitro and in silico methods. In vitro studies revealed that the derivatives (**8a**–**k**) showed significant inhibition activity. The results showed that the novel derivatives (**8a**–**k**) demonstrated potential inhibitory activity, with *K*_I_ values covering the following ranges: 23.47 ± 2.40 to 139.60 ± 13.33 nM for AR and 6.09 ± 0.37 to 119.80 ± 12.31 μM for α-GLY, with IC_50_ values 81.14 to 153.51 μM for α-AMY. Furthermore, many of these compounds exhibited high inhibition activity, while some of them showed higher potency than the reference compounds. Molecular docking of the target compounds was carried out in the active sites of AR (PDB ID: 4JIR) and α-GLY (PDB ID: 5NN8).

## 1 Introduction

Diabetes mellitus (DM) is a widespread, multifactorial chronic health condition corresponding with prolonged high blood sugar levels. Type 2 DM (T2DM) is a noninsulin-dependent type of DM and is characterized by defective insulin action associated with various complications, including cardiovascular diseases, kidney failure, neuronal diseases, diabetic retinopathy, and hyperlipidemia.^1,2^ 642 million people are globally expected to be affected by DM by the year 2040.^3^

Hyperglycemia is the most critical risk factor related to the progression of DM. Hence, various oral antihyperglycemic drugs with different mechanisms of action have been developed to keep glycaemia under control in the prevention of long-term diabetes complications, e.g., cataracts, neuropathy, retinopathy, nephropathy, and several cardiovascular diseases.^4^ One of the therapeutic approaches to controlling glucose concentration is to inhibit several digestive enzymes that result in preventing carbohydrate digestion and reducing their absorption.^5^ Intestinal α-glucosidase or -glycosidase (α-GLY) catalyzes the hydrolysis of 1,4-α-glycosidic linkages in disaccharides and oligosaccharides, converting them to monosaccharides for absorption.^6^ Inhibitors of α-GLY are approved as antihyperglycemic drugs since such molecules reduce postprandial plasma glucose concentrations by delaying the release of glucose in the intestine lumen.^7^

Similar to α-GLY, pancreatic α-amylase (α-AMY) is another key enzyme in the digestive system that is responsible for breaking down long-chain carbohydrates to glucose, therefore becoming another target in the management of T2DM.^8,9^ Therefore, the inhibition of α-GLY and α-AMY enzymes may be a potential treatment strategy for T2DM. Aldose reductase (AR) is an NADPH-dependent oxidoreductase that catalyzes the reduction of glucose to sorbitol in the polyol pathway. At a high concentration of glucose, 30% of the glucose enters into the polyol pathway, leading to the overproduction of sorbitol.^10,11^ Due to its hydrophilic nature, sorbitol cannot easily diffuse across the plasma membranes and accumulates inside the cells in the various tissues, such as the lens, retina, kidney, and peripheral nerves, thus inducing osmotic stress, which is associated with the complications of diabetes.^12,13^ Therefore, AR inhibitors play a crucial role in the pathogenesis of diabetic complications in various tissues.

In the last decades, α-GLY inhibitors named acarbose, miglitol, and voglibose have been clinically approved in the management of T2DM. The undesirable adverse gastrointestinal effects of these carbohydrate mimic-based α-GLY inhibitors have limited their clinical applications.^14,15^ In the last years, researchers showed an extensive effort to discover synthetic α-GLY inhibitors with better efficacy and minimal side effects. Among them, 1,3,4-thiadiazole-based compounds were recently introduced as potent α-GLY inhibitors.^16−22^ 1,3,4-Thiadiazole compounds have also been reported to interact with various targets, including α-AMY inhibitor,^23^ FFA1/PPARδ agonist,^24^ sodium-dependent glucose cotransporter-2 (SGLT-2) inhibitor,^25^ c-Jun N-terminal kinase inhibitor,^23^ and cannabinoid-1 receptor antagonist^24^ in the management of T2DM. Imidazopyridine is also an important N-containing heterocyclic scaffold in medicinal chemistry. Imidazopyridine derivatives have been declared to possess antidiabetic activity through various targets: GSK3β inhibitor,^26^ fatty acid synthase inhibitor,^27^ GPR40 agonist,^28^ α-GLY inhibitor,^29^ α-AMY inhibitor,^30^ and DPP-4 inhibitor.^31^

In this study, we aimed to design and synthesize hybrid analogues of 1,3,4-thiadiazole and imidazo[1,2-*a*]pyridine to obtain potential inhibitors of AR, α-GLY, and α-AMY. All the synthesized compounds were characterized by ^1^H NMR, ^13^C NMR, and HRMS. Molecular docking studies of most potent compounds were also conducted to corroborate the observed enzyme inhibitory activities.

## 2 Results and Discussion

### 2.1 Chemistry

**Scheme 1:**
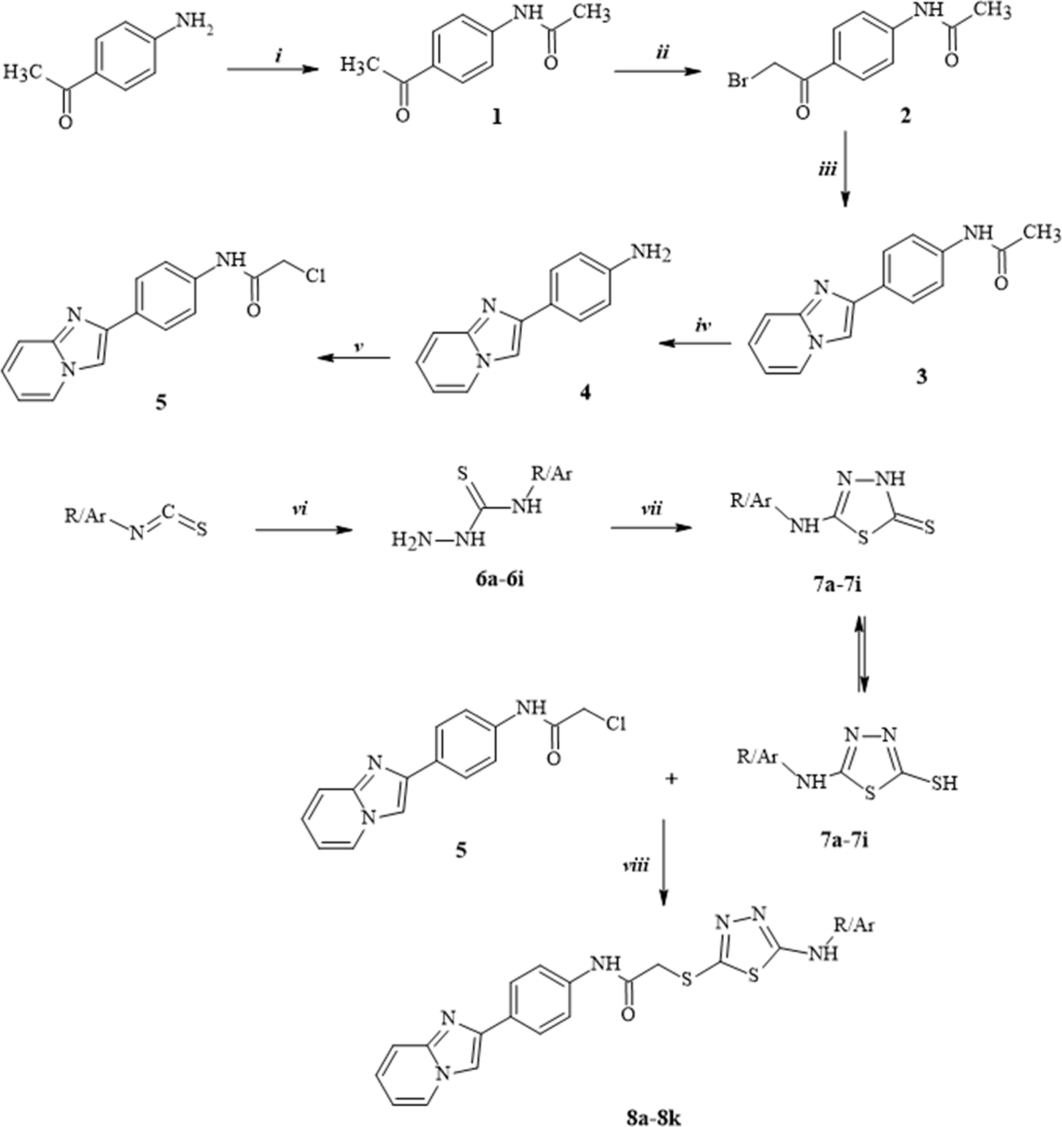
Synthetic Protocol of Compounds (**8a**–**k**) Reagents, conditions, and yield: (i) CH_3_COCl, Et_3_N, THF, ice bath, 4 h, 70%; (ii) Br_2_, HBr, AcOH, ice bath, 3 h, 75%; (iii) 2-aminopyridine, EtOH, reflux, 2 h; 67%; (iv) % 10 HCl, reflux, 2 h, 76%; (v) ClCOCH_2_Cl, Et_3_N, THF, ice bath, 4 h, 65%; (vi) NH_2_NH_2_·H_2_O, EtOH, r.t., 4 h; (vii) (1) CS_2_/KOH, EtOH, reflux, 10 h; (2) HCl, pH 4–5; (viii) K_2_CO_3_, acetone, r.t., 8 h.

Target compounds were synthesized via an eight-step synthetic strategy depicted in Scheme 1. The starting material 1-(4-aminophenyl)ethan-1-one was acetylated with acetyl chloride in the presence of triethylamine to synthesize compound **1**. Furthermore, α-bromination of compound **1** led to the formation of compound **2**, which then reacted with 2-aminopyridine to obtain compound **3** via a ring closure reaction. Then, the hydrolysis of the acetyl group with 10% HCl led to the formation of compound **4**. Compound **5** was synthesized through the reaction with compound **4** and chloroacetyl chloride in the presence of triethylamine. The reaction conducted between various substituted isothiocyanates and hydrazine hydrate afforded compounds **6a**–**6i**, which then reacted with carbon disulfide to give 5-substituted amino-1,3,4-thiadiazole-2(3*H*)-thiones (**7a**–**7i**). In the last step, derivatives of compound **5** and **7a**–**7i** were reacted in the presence of potassium carbonate to prepare the desired products.

The structures of compounds **8a**–**k** were confirmed using techniques such as NMR and HRMS. In the ^1^H NMR spectra of all compounds, the singlet peaks due to two NH proton and –COCH_2_ protons were detected in between 9.80 and 10.55 and 3.55–4.16 ppm, respectively. A singlet peak at 3.71–3.84 ppm proved the existence of methoxy protons in compounds **8h**, **8i**, and **8k**. In the spectra of compound **8e**, the singlet peak at 2.24 ppm belongs to the *p*-methyl substituent on the phenyl ring. In the spectra of compound **8b**, a triplet peak at 1.14 ppm and a quartet peak at 2.53 ppm were assigned to the *p*-ethyl group bonded to the phenyl ring. The other aromatic and aliphatic protons were observed in the expected regions for all compounds.

In the ^13^C NMR spectra of the compounds, the peaks related to the CH_2_ carbon attached to the carbonyl group and carbonyl carbon were recorded between 38.72 and 39.09 and 163.82–169.44 ppm, respectively. The other aromatic and aliphatic carbons were observed in expected regions.

### 2.2 Biological Activity

Heterocyclic compounds containing nitrogen, sulfur, and oxygen heteroatoms play a critical role in many biochemical processes that are important for life. These compounds constitute the most important class of compounds in the pharmaceutical and agrochemical industries and comprise about 60% of drug substances. Five-membered heterocycles containing nitrogen and oxygen or sulfur, e.g., isothiazole, oxadiazole, oxazolidine, thiazole, thiazolidine, isothiazolidine, and thiadiazole, are important structural motifs. Such compounds are found in a large number of biologically active molecules and are of great importance for drug discovery and development in the pharmaceutical industry, as they form the central core of many therapeutic agents.^32,33^ Selective functionalization of functional compounds with bioactivity using various substituents enhances their efficacy in diverse fields. These individual and different properties of thiadiazole-derived compounds have directed attention to the design of alternative thiadiazole derivatives with varying selectivity toward diabetes disease-related enzymes.

In the scholarly literature, numerous studies have investigated the synthesis of thiadiazole and imidazopyridine derivatives and their influence on diabetes disease-related enzymes such as AR, α-GLY, and α-AMY.^24,29,34,35^ Based on previous studies targeting multiple enzymes,^36−39^ our current study aims to investigate the inhibitory effects of newly designed and synthesized imidazo[1,2-*a*]pyridine-based 1,3,4-thiadiazole derivatives (**8a**–**k**) on the AR, α-GLY, and α-AMY. The research protocols were carried out by spectrophotometric methods developed by Tahtah,^40^ Tao,^41^ and Xiao,^42^ which provide high reliability and accuracy for the evaluation of AR, α-GLY, and α-AMY inhibition. The findings and inhibition data are presented in detail in Table 1. In the study, clinically recognized inhibitors, namely, epalrestat (for AR inhibition) and acarbose (for α-GLY and α-AMY inhibition), were used as reference standards, and a comparative analysis of the results was provided.

**Table 1:** Inhibitory Effects of the Novel Imidazo[1,2-*a*]pyridine-Based 1,3,4-Thiadiazole Derivatives (**8a**–**k**) on AR, α-GLY, and α-AMY^b^

		AR^a^		α-GLY^c^		α-AMY^d^	
comp.	R/Ar	*K*_I_ (nM)	*R*^2^	*K*_I_ (μM)	*R*^2^	IC_50_ (μM)	*R*^2^
**8a**	phenyl	34.91 ± 3.59	0.9857	33.04 ± 1.25	0.9877	141.75	0.9856
**8b**	4-ethylphenyl	74.16 ± 8.01	0.9851	**6.09±0.37**	0.9864	113.03	0.9765
**8c**	cyclohexyl	139.60 ± 13.33	0.9874	77.50 ± 5.74	0.9866	N.D^d^	
**8d**	ethyl	78.53 ± 8.24	0.9845	88.65 ± 17.72	0.9834	121.80	0.9684
**8e**	4-methylphenyl	44.29 ± 4.15	0.9878	**13.03±2.43**	0.9737	N.D^d^	
**8f**	4-chlorophenyl	**23.47±2.40**	0.9863	31.15 ± 2.29	0.9871	141.99	0.9756
**8g**	3-chlorophenyl	71.35 ± 7.29	0.9859	38.03 ± 6.06	0.9871	131.85	0.9823
**8h**	3-methoxyphenyl	45.35 ± 2.94	0.9873	43.40 ± 3.17	0.9863	143.27	0.9698
**8i**	2-methoxyphenyl	80.32 ± 8.02	0.9866	**15.68±1.97**	0.9889	153.51	0.9785
**8j**	2-chlorophenyl	69.87 ± 7.17	0.9858	119.80 ± 12.31	0.9862	81.14	0.9664
**8k**	4-methoxyphenyl	97.78 ± 10.30	0.9865	48.87 ± 7.11	0.9886	112.57	0.9816
**epalrestat**		828.70 ± 60.42	0.9841				
**acarbose**				24.36 ± 3.12	0.9723	54.37	0.9842

a Aldose reductase.

b α-Glycosidase.

c α-Amylase.

d Not determined.

The synthesized derivatives (**8a**–**k**) have nanomolar and micromolar inhibitory activities on tested enzymes. The inhibition potential of these derivatives for AR was determined with *K*_I_ values ranging from 23.47 ± 2.40 to 139.60 ± 13.33 nM. In particular, the newly developed derivatives exhibited superior inhibition activity on AR compared to the reference compound epalrestat (*K*_I_; 828.70 ± 60.42 nM). Among the synthesized compounds, *para*-chlorophenyl derivative **8f** was the most potent inhibitor against AR with 23.47 ± 2.40 nM. The replacement of hydrogen with bulkier groups both electron-withdrawing and electron-donating in the phenyl (**8a**) or substituted phenyl bearing compounds (**8b**, **8e**, **8f**, **8g**, **8h**, **8i**, **8j**, and **8k**) effected AR inhibitory activity at varying degrees. Among chlorophenyl moiety-containing derivatives, the AR inhibitory activity reduced in the order of **8f** (*para*-chloro) > **8j** (*ortho*-chloro) > **8g** (*meta*-chloro). The AR inhibition of methoxyphenyl derivatives was observed to increase in order of **8k** (*para*-methoxy) < **8i** (*ortho*-methoxy) < **8h** (*meta*-methoxy). On the other hand, compound **8c** exhibited an inhibitory effect with a *K*_I_ value of 139.60 ± 13.33 nM, higher than the standard compound but lower than the other derivatives. Among compounds that substitute with aliphatic groups, it can be claimed that the ethyl moiety (**8d**) increased the AR inhibitory activity more than the cyclohexyl moiety (**8c**). In enzyme kinetics, *K*_I_ values are known to indicate the affinity and selectivity of the inhibitor for the enzyme. Considering this situation, it was observed that compound **8c** exhibited the lowest selectivity for AR, whereas **8f** showed the highest selectivity (Table 1). In a study, 5-benzyl-2,4-thiazolidinedione derivatives were synthesized and tested as AR (ALR2) inhibitors in vitro. These compounds exhibited an inhibition effect on the enzyme at the micromolar level, with IC_50_ values in the range of 1.07–78.90 μM.^43^ In another study, thiazole-based compounds were synthesized. These compounds showed inhibitory potential effect against AR with *K*_I_ values in the range of 0.018 ± 0.005 μM to 3.746 ± 1.321 μM.^44^ The imidazo[1,2-*a*]pyridine-based 1,3,4-thiadiazole compounds synthesized in the study showed a more effective AR inhibition effect at the micromolar level than the potential inhibitors mentioned above.

The synthesized compounds showed inhibitory potential with *K*_I_ values ranging from 6.09 ± 0.37 to 119.80 ± 12.31 μM for α-GLY and IC_50_ values ranging from 81.14 to 153.51 μM for α-AMY. In particular, some of the tested compounds (**8b**, **8e**, and **8i**) showed higher inhibition against α-GLY than the reference compound acarbose (*K*_I_ = 24.36 ± 3.12). Among these compounds, compounds **8b** and **8e** showed a very strong inhibition effect against the α-GLY enzyme with a remarkable *K*_I_ value of 6.09 ± 0.37 μM (noncompetitive inhibition) and 13.03 ± 2.43 μM (competitive inhibition), respectively. In contrast, compound **8j** showed lower inhibition activity than the others, with a *K*_I_ value of 119.80 ± 12.31 μM. According to results, it is suggested that electron-donating substituents ethyl (**8b**) and methyl (**8e**) on the phenyl ring are the most favorable for the α-GLY inhibitory activity. Except for this, the methoxy group at the *ortho* position (**8i**) enhanced the activity the most in comparison with the derivatives containing other electron-withdrawing substituents.

For α-AMY, *ortho*-chlorophenyl derivative **8j** exhibited higher inhibitory potential than the other compounds, with an IC_50_ value of 81.14 μM. In contrast, *ortho*-methoxyphenyl derivative **8i** showed a weaker potential inhibitory effect with an IC_50_ value of 153.51 μM compared to the others (Table 1).

The results indicate that the variations in the inhibitory activity of thiadiazole derivatives against AR, α-GLY, and α-AMY enzymes may depend on the specific substitution pattern of R. The deviations in inhibitory potency observed in the analogues can be attributed to various functional groups in the variable substitution pattern of R on the derivatives.

### 2.3 Molecular Docking Study

Utilizing the X-ray crystallographic structures of the AR (PDB ID: 4JIR)^45^ and α-GLY (PDB ID: 5NN8),^46^ the binding patterns of the newly synthesized imidazo[1,2-*a*]pyridine-based 1,3,4-thiadiazoles (**8a**–**k**) were evaluated. To validate the docking setup, the native ligands, epalrestat, and acarbose, respectively, were redocked into the enzyme binding sites. The RMSD values are <1.0, and the ability of the docking poses to recapitulate all critical interactions confirmed the reliability of the docking methodology employed for this study. In this protocol, the proteins were kept rigid, while the ligands were allowed to be flexible throughout the docking simulation.

**Figure 1:**
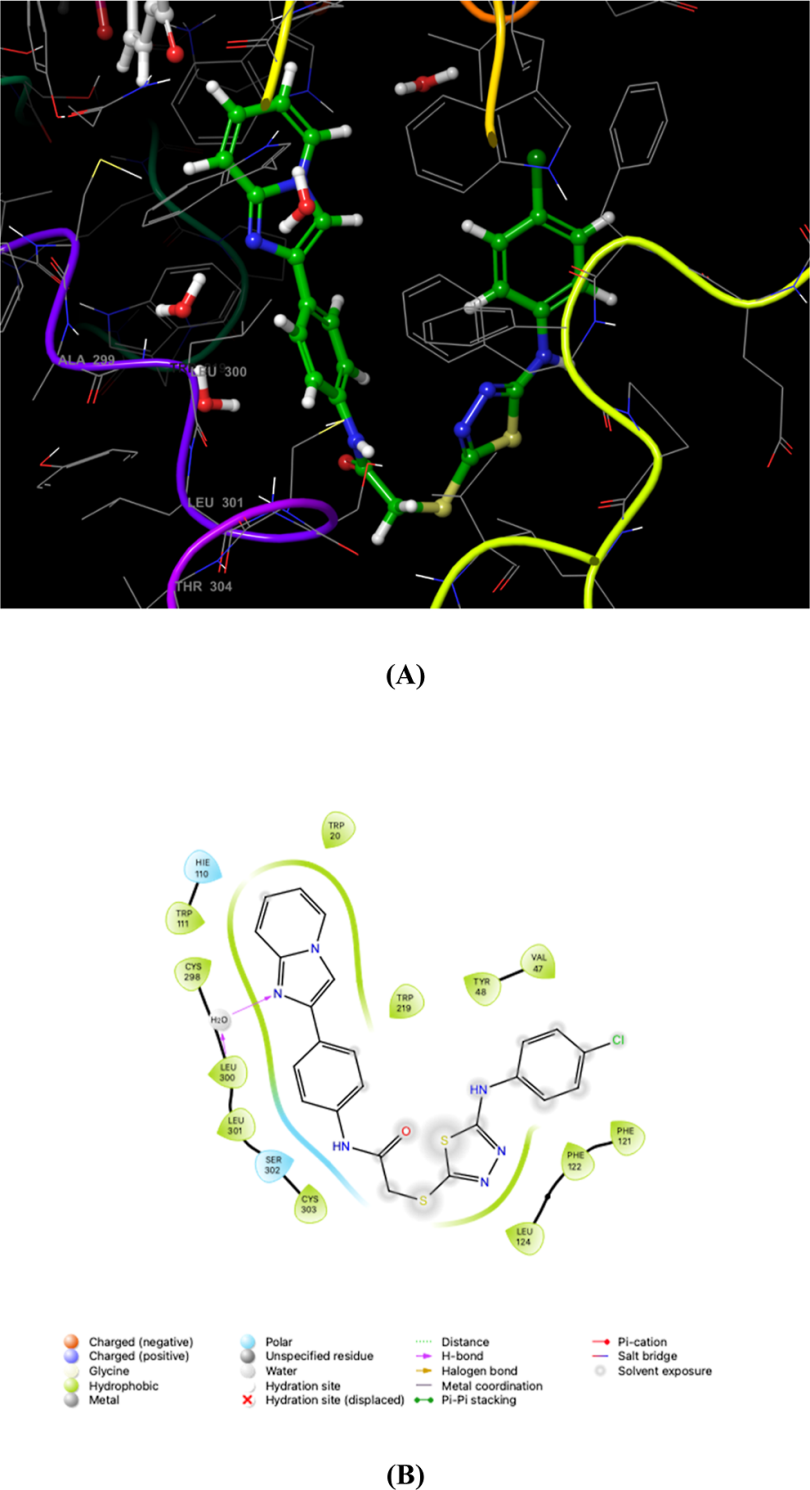
AR, represented by PDB ID: 4JIR, was subjected to molecular docking studies with 2-[(5-(4-chlorophenyl)amino-1,3,4-thiadiazol-2-yl)thio]-*N*-[4-(imidazo[1,2-*a*]pyridin-2-yl)phenyl] acetamide (**8f**). The resulting 3D docking conformation of compound **8f** within the 4JIR binding pocket is illustrated in panel A. 2D interaction diagram is presented in panel B to delineate the molecular interactions further, highlighting the interactions between 4JIR and compound **8f**.

Upon binding to the active site of AR, this class of inhibitors induces a conformational change that forms a pocket between Trp111 and Leu300.^47^ This conformational adaptation, “induced fit”, allows the pocket to adjust and accommodate each specific inhibitor. The residues forming this pocket are unique to AR and ensure the specificity of interactions for AR. Consequently, inhibitors engaging with this “specificity pocket” exhibit high selectivity for AR. For the most potent inhibitor in this series, compound 8f, the specific interaction within the specificity pocket includes an almost ideal hydrogen bond formed the water-mediated between Leu300 and the imidazole ring (Figure 1).

**Figure 2:**
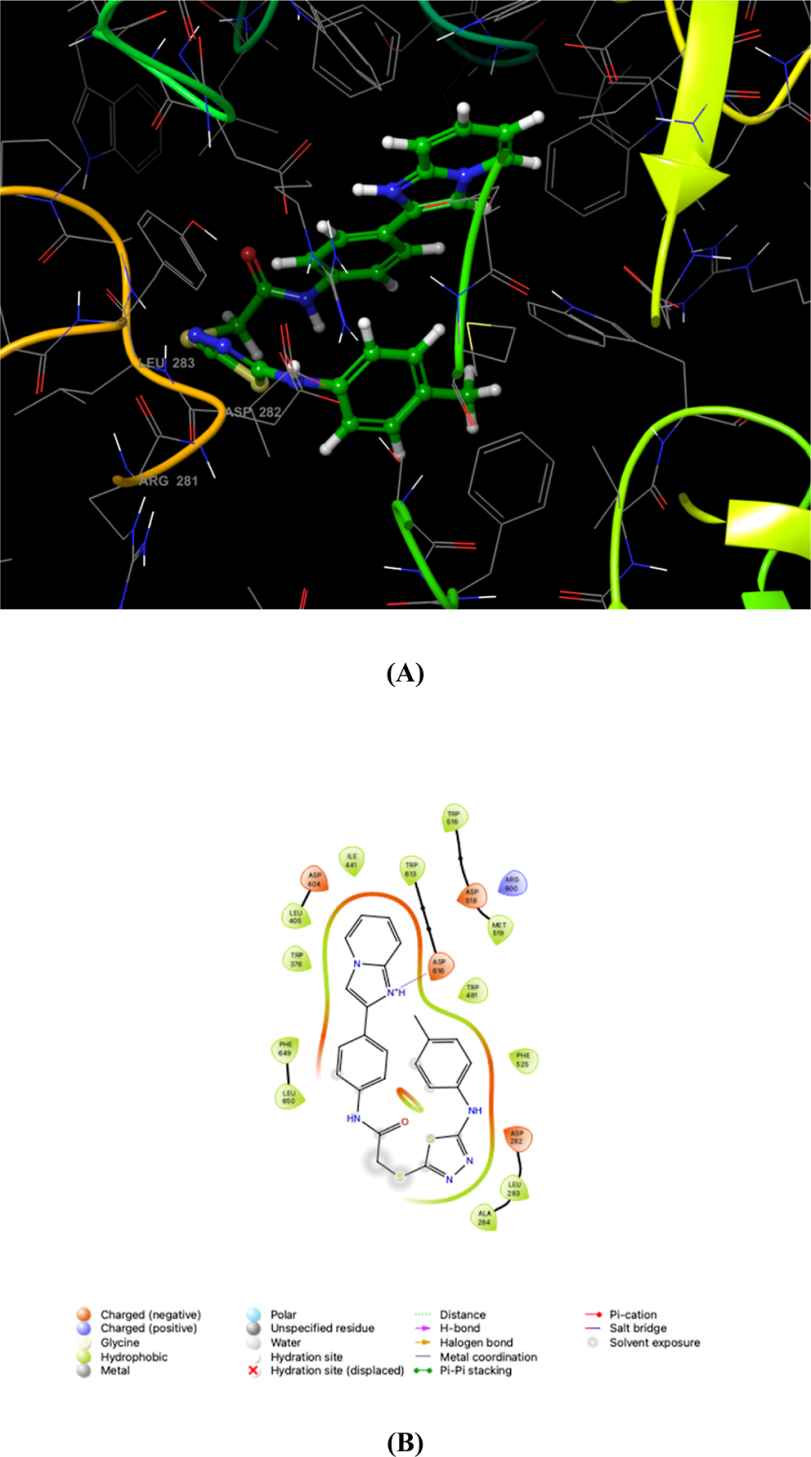
α-Glycosidase (α-GLY), represented by PDB ID: 5NN8, was subjected to molecular docking studies with 2-[(5-(4-methylphenyl)amino-1,3,4-thiadiazol-2-yl)thio]-*N*-[4-(imidazo[1,2-*a*]pyridin-2-yl)phenyl] acetamide (**8e**). The resulting 3D docking conformation of compound **8e** within the 5NN8 binding pocket is illustrated in panel A. 2D interaction diagram is presented in panel B to delineate the molecular interactions further, highlighting the interactions between 5NN8 and compound **8e**.

The maltose moiety of acarbose, situated in subsites +2 and +3, does not engage directly with α-GLY but is instead stabilized by crystal lattice packing interactions and a water-mediated contact with the side chain of Trp618, which is positioned at the edge of the substrate-binding pocket. Notably, compound **8e**, the most potent inhibitor, establishes a salt bridge with the Asp616 residue. This limited number of interactions indicates that α-GLY has only two effective substrate-binding sites (Figure 2).

In conclusion, the inhibition data obtained in this study reveal that the new thiadiazole derivatives exhibit remarkable inhibitory potential on tested enzymes in comparison with various compounds in the literature. These results emphasize the need for not only further investigations into the mechanisms of enzyme inhibition but also structural modifications for the development of new therapeutic agents for the treatment of diabetes. Thus, these findings increase the potential of new thiadiazole derivatives as promising AR, α-GLY, and α-AMY inhibitors that may offer important contributions in terms of potential applications in the management of DM.

## 3 Conclusions

All synthesized imidazo[1,2-*a*]pyridine-based 1,3,4-thiadiazole derivatives (**8a**–**k**) were evaluated for their AR, α-GLY, and α-AMY inhibitory effects and exhibited more potent inhibition in the range of *K*_I_ = 23.47 ± 2.40 to 139.60 ± 13.33 nM for AR than the standard epalrestat (*K*_I_ = 828.70 ± 60.42 nM). Compounds **8b**, **8e**, and **8i** showed higher inhibition with *K*_I_ values of 6.09 ± 0.37 μM, 13.03 ± 2.43 μM, and 15.68 ± 1.97 μM, respectively, against α-GLY than the reference compound acarbose (*K*_I_ = 24.36 ± 3.12). Moreover, a molecular docking study was conducted to understand the ligand–enzyme interactions. Considering the obtained results, the current study has identified a series of lead molecules as multitarget inhibitors of AR and α-GLY for advanced research in order to obtain drugs to be used in the treatment of diabetes.

## 4 Materials and Methods

### 4.1 Chemistry

#### 4.1.1 Preparation of *N*-(4-Acetylphenyl)acetamide (**1**)

1-(4-Aminophenyl)ethan-1-one (0.11 mol, 15 g) was dissolved in tetrahydrofuran (250 mL), and triethylamine (0.13 mol, 18.56 mL) was added. The mixture was cooled in an ice bath, and acetyl chloride (0.13 mol, 9.5 mL) was added dropwise with stirring. After addition of acetyl chloride, the reaction mixture was stirred for an additional 1 h at room temperature. The solvent was evaporated under reduced pressure, and the product was washed with water, dried, and recrystallized from ethanol.

#### 4.1.2 Preparation of *N*-[4-(2-Bromoacetyl)phenyl]acetamide (**2**)

Compound **1** (0.1 mol, 17.9 g) was dissolved in acetic acid (250 mL), and a catalytic amount of hydrobromic acid (3 mL) was added. The mixture was cooled in an ice bath, and bromine (0.12 mol, 6.3 mL) was added dropwise with stirring. After TLC screening, the reaction mixture was poured into ice water, and the precipitated product was filtered and recrystallized from ethanol.

#### 4.1.3 Preparation of *N*-[4-(Imidazo[1,2-*a*]pyridin-2-yl)phenyl]acetamide (**3**)

Compound **2** (78 mmol) and 2-aminopyridine (78 mmol) were refluxed in ethanol (250 mL) for 2 h. After the completion of the reaction, the product was filtered and recrystallized from ethanol.

#### 4.1.4 Preparation of 4-(Imidazo[1,2-*a*]pyridin-2-yl)aniline (**4**)

Compound **3** (64 mmol) was refluxed with a % 10 HCl solution in water (200 mL). After TLC screening, the mixture was poured into ice water and neutralized with ammonia. The precipitated product was filtered and recrystallized from ethanol.

#### 4.1.5 Preparation of 2-Chloro-*N*-[4-(imidazo[1,2-*a*]pyridin-2-yl)phenyl]acetamide (**5**)

Compound **4** (53 mmol) was dissolved in tetrahydrofuran (200 mL), and triethylamine (63.6 mmol, 8.86 mL) was added. In an ice bath, chloroacetyl chloride (63.6 mmol, 5.06 mL) was added dropwise with stirring. After the completion of dripping, the solvent was evaporated under reduced pressure, and the product was washed with water, dried, and recrystallized from ethanol.

#### 4.1.6 General Preparation of 4-Substituted Thiosemicarbazides (**6a–6i**)

A mixture of substituted isothiocyanate (20 mmol) and hydrazine hydrate (40 mmol) in ethanol (50 mL) was stirred at room temperature for 4 h. The precipitated compound was filtered and crystallized from ethanol.

#### 4.1.7 General Preparation of 5-Substituted Amino-1,3,4-thiadiazole-2(3*H*)-thiones (**7a–7i**)

Carbon disulfide (27 mmol, 1.6 mL) was added to a solution of 4-substituted thiosemicarbazides (**6a**–**6i**) and potassium hydroxide in ethanol, and the mixture was refluxed for 10 h. The solution was cooled and acidified to pH 4–5 with a hydrochloric acid solution and crystallized from ethanol.

#### 4.1.8 General Preparation of *N*-[4-(Imidazo[1,2-*a*]pyridin-2-yl)phenyl]-2-[(5-substituted-1,3,4-thiadiazole)acetamide Derivatives (**8a–8k**)

A mixture of 5-substituted amino-1,3,4-thiadiazole-2(3*H*)-thione (**7a**–**7i**) (4 mmol) and 2-chloro-*N*-[4-(imidazo[1,2-*a*]pyridin-2-yl)phenyl]acetamide (**5**) (4 mmol) in acetone (40 mL) was stirred at room temperature for 8 h in the presence of potassium carbonate (5 mmol, 0.66 g). After the evaporation of acetone, the residue was washed with water and crystallized from ethanol.

##### 4.1.8.1 2-[(5-Phenylamino-1,3,4-thiadiazol-2-yl)thio]-*N*-[4-(imidazo[1,2-*a*]pyridin-2-yl)phenyl] Acetamide (**8a**)

Last step yield: 72%, mp = 223.0 °C. ^1^H NMR (300 MHz, DMSO-*d*_6_): δ = 4.15 (2H, s, CH_2_), 6.90 (1H, t, *J* = 6.69 Hz, imidazo[1,2-*a*]pyridine–CH), 6.99 (1H, t, *J* = 7.32 Hz, benzene–CH), 7.23–7.28 (1H, m, imidazo[1,2-*a*]pyridine–CH), 7.34 (2H, t, *J* = 7.53 Hz, benzene–CH), 7.57 (3H, d, *J* = 8.67 Hz, benzene–CH, imidazo[1,2-*a*]pyridine–CH), 7.67 (2H, d, *J* = 8.67 Hz, 1,4-disubstituted benzene–CH), 7.93 (2H, d, *J* = 8.64 Hz, 1,4-disubstituted benzene–CH), 8.35 (1H, s, imidazo[1,2-*a*]pyridine–CH), 8.52 (1H, d, *J* = 6.75 Hz, imidazo[1,2-*a*]pyridine–CH), 10.41 (1H, s, NH), 10.43 (1H, s, NH). ^13^C NMR (75 MHz, DMSO-*d*_6_): 38.96 (C), 109.10 (C), 112.78 (C), 116.84 (C), 117.25 (C), 117.88 (2C), 119.80 (2C), 121.48 (C), 122.49 (C), 125.51 (C), 126.59 (2C), 127.33 (C), 129.44 (C), 129.59 (2C), 138.86 (C), 140.84 (C), 145.13 (C), 165.61 (C), 166.11 (C). HRMS (*m*/*z*): [M + H]^+^ calcd for C_23_H_18_N_6_OS_2_, 459.1056; found, 459.1044.

##### 4.1.8.2 2-[(5-(4-Ethylphenyl)amino-1,3,4-thiadiazol-2-yl)thio]-*N*-[4-(imidazo[1,2-*a*]pyridin-2-yl) phenyl]acetamide (**8b**)

Last step yield: 74%, mp = 244.2 °C. ^1^H NMR (300 MHz, DMSO-*d*_6_): δ = 1.14 (3H, t, *J* = 7.59 Hz, CH_3_), 2.53 (2H, q, *J* = 7.53 Hz, CH_2_), 4.13 (2H, s, CH_2_), 6.88 (1H, t, *J* = 6.72 Hz, imidazo[1,2-*a*]pyridine–CH), 7.15 (2H, d, *J* = 8.46 Hz, 1,4-disubstituted benzen–CH), 7.24 (1H, t, *J* = 6.72 Hz, imidazo[1,2-*a*]pyridine–CH), 7.45 (2H, d, *J* = 8.52 Hz, 1,4-disubstituted benzen–CH), 7.54–7.57 (1H, m, imidazo[1,2-*a*]pyridine–CH), 7.66 (2H, d, *J* = 8.70 Hz, 1,4-disubstituted benzen–CH), 7.92 (2H, d, *J* = 8.64 Hz, 1,4-disubstituted benzen–CH), 8.34 (1H, s, imidazo[1,2-*a*]pyridine–CH), 8.51 (1H, d, *J* = 6.75 Hz, imidazo[1,2-*a*]pyridine–CH), 10.32 (1H, s, NH), 10.43 (1H, s, NH). ^13^C NMR (75 MHz, DMSO-*d*_6_):16.21 (C), 27.96 (C), 38.96 (C), 109.06 (C), 109.10 (C), 112.71 (C), 116.89 (C), 118.02 (2C), 119.76 (2C), 125.41 (C), 126.57 (2C), 127.30 (C), 128.79 (2C), 129.65 (C), 137.98 (C), 138.63 (C), 138.83 (C), 144.50 (C), 145.18 (C), 152.08 (C), 166.13 (C). Anal. Calcd For C_25_H_22_N_6_OS_2_: C, 61.71; H, 4.56; N, 17.27. Found: C, 61.80; H, 4.55; N, 17.31. HRMS (*m*/*z*): [M + 2H]^+2^/2 calcd for C_25_H_22_N_6_OS_2_, 244.0721; found, 244.0712.

##### 4.1.8.3 2-[(5-Cyclohexylamino-1,3,4-thiadiazol-2-yl)thio]-*N*-[4-(imidazo[1,2-*a*]pyridin-2-yl)phenyl] Acetamide (**8c**)

Last step yield: 69%, mp = 199.8 °C. ^1^H NMR (300 MHz, DMSO-*d*_6_): δ = 1.18–1.30 (5H, m, cyclohexyl CH), 1.52–1.55 (1H, m, cyclohexyl CH), 1.65–1.69 (2H, m, cyclohexyl CH), 1.90–1.94 (2H, m, cyclohexyl CH), 3.17 (1H, s, cyclohexyl CH), 4.00 (2H, s, CH_2_), 6.88 (1H, t, *J* = 6.72 Hz, imidazo[1,2-*a*]pyridine–CH), 7.24 (1H, t, *J* = 6.87 Hz, imidazo[1,2-*a*]pyridine–CH), 7.55 (1H, d, *J* = 9.30 Hz, imidazo[1,2-*a*]pyridine–CH), 7.65 (2H, d, *J* = 8.70 Hz, 1,4-disubstituted benzen–CH), 7.91 (2H, d, *J* = 8.61 Hz, 1,4-disubstituted benzen–CH), 8.33 (1H, s, imidazo[1,2-*a*]pyridine–CH), 8.51 (1H, d, *J* = 6.75 Hz, imidazo[1,2-*a*]pyridine–CH), 10.36 (1H, s, NH). ^13^C NMR (75 MHz, DMSO-*d*_6_): 24.66 (2C), 25.66 (C), 32.46 (2C), 39.06 (C), 53.92 (C), 109.04 (C), 112.66 (C), 116.95 (C), 119.78 (2C), 125.31 (C), 126.54 (2C), 127.28 (C), 127.62 (C), 128.71 (C), 129.74 (C), 138.81 (C), 144.62 (C), 166.28 (C), 169.47 (C). Anal. Calcd For C_23_H_24_N_6_OS_2_: C, 59.46; H, 5.21; N, 18.09. Found: C, 59.56; H, 5.20; N, 18.12. HRMS (*m*/*z*): [M + H]^+^ calcd for C_23_H_24_N_6_OS_2_, 465.1526; found, 465.1527.

##### 4.1.8.4 2-[(5-Ethylamino-1,3,4-thiadiazol-2-yl)thio]-*N*-[4-(imidazo[1,2-*a*]pyridin-2-yl)phenyl] Acetamide (**8d**)

Last step yield: 76%, mp = 191.1 °C. ^1^H NMR (300 MHz, DMSO-*d*_6_): δ = 1.17 (3H, t, *J* = 7.26 Hz, CH_3_), 3.07 (2H, q, *J* = 4.71 Hz, CH_2_), 4.24 (2H, s, CH_2_), 6.89–6.93 (3H, m, imidazo[1,2-*a*]pyridine–CH, benzene–CH), 7.08 (1H, d, *J* = 8.94 Hz, imidazo[1,2-*a*]pyridine–CH), 7.30–7.33 (3H, m, imidazo[1,2-*a*]pyridine–CH, benzene–CH), 7.45 (2H, d, *J* = 9.03 Hz, 1,4-disubstituted benzen–CH), 10.20 (1H, s, NH). ^13^C NMR (75 MHz, DMSO-*d*_6_): 15.60 (C), 35.45 (C), 37.46 (C), 114.75 (C), 114.80 (2C), 115.15 (C), 117.03 (C), 117.96 (C), 119.70 (2C), 120.59 (C), 125.94 (C), 129.87 (C), 133.56 (C), 134.23 (C), 149.50 (C), 160.48 (C), 167.14 (C). Anal. Calcd For C_19_H_18_N_6_OS_2_: C, 55.59; H, 4.42; N, 20.47. Found: C, 55.74; H, 4.41; N, 20.51. HRMS (*m*/*z*): [M + 2H]^+2^/2 calcd for C_19_H_18_N_6_OS_2_, 206.0565; found, 206.0556.

##### 4.1.8.5 2-[(5-(4-Methylphenyl)amino-1,3,4-thiadiazol-2-yl)thio]-*N*-[4-(imidazo[1,2-*a*]pyridin-2-yl) phenyl]acetamide (**8e**)

Last step yield: 70%, mp = 130.1 °C. ^1^H NMR (300 MHz, DMSO-*d*_6_): δ = 2.27 (3H, s, CH_3_), 4.12 (2H, s, CH_2_), 6.90 (1H, t, *J* = 6.72 Hz, imidazo[1,2-*a*]pyridine–CH), 7.04 (1H, t, *J* = 7.41 Hz, benzene–CH), 7.18–7.28 (3H, m, benzene–CH, imidazo[1,2-*a*]pyridine–CH), 7.57 (1H, d, *J* = 8.01 Hz, imidazo[1,2-*a*]pyridine–CH), 7.66 (2H, d, *J* = 8.70 Hz, 1,4-disubstituted benzene–CH), 7.81 (1H, d, *J* = 7.74 Hz, benzene–CH), 7.93 (2H, d, *J* = 8.64 Hz, 1,4-disubstituted benzene–CH), 8.35 (1H, s, imidazo[1,2-*a*]pyridine–CH), 8.52 (1H, d, *J* = 6.72 Hz, imidazo[1,2-*a*]pyridine–CH), 10.40 (1H, s, NH). ^13^C NMR (75 MHz, DMSO-*d*_6_): 18.38 (C), 38.85 (C), 109.09 (C), 112.66 (C), 116.96 (C), 119.80 (2C), 121.69 (2C), 124.49 (2C), 125.31 (C), 126.56 (2C), 127.12 (C), 127.27 (C), 129.49 (C), 129.78 (C), 131.17 (C), 137.49 (C), 138.79 (C), 144.64 (C), 166.16 (C), 167.85 (C). Anal. Calcd For C_24_H_20_N_6_OS_2_: C, 60.99; H, 4.27; N, 17.78. Found: C, 61.08; H, 4.26; N, 17.82. HRMS (*m*/*z*): [M + 2H]^+2^/2 calcd for C_24_H_20_N_6_OS_2_, 237.0643; found, 237.0630.

##### 4.1.8.6 2-[(5-(4-Chlorophenyl)amino-1,3,4-thiadiazol-2-yl)thio]-*N*-[4-(imidazo[1,2-*a*]pyridin-2-yl) phenyl]acetamide (**8f**)

Last step yield: 76%, mp = 265.0 °C. ^1^H NMR (300 MHz, DMSO-*d*_6_): δ = 4.16 (2H, s, CH_2_), 6.88 (1H, t, *J* = 6.81 Hz, imidazo[1,2-*a*]pyridine–CH), 7.24 (1H, t, *J* = 7.86 Hz, imidazo[1,2-*a*]pyridine–CH), 7.37 (2H, d, *J* = 8.94 Hz, 1,4-disubstituted benzene–CH), 7.57–7.60 (3H, m, imidazo[1,2-*a*]pyridine–CH, benzene–CH), 7.66 (2H, d, *J* = 8.73 Hz, 1,4-disubstituted benzene–CH), 7.92 (2H, d, *J* = 8.61 Hz, 1,4-disubstituted benzene–CH), 8.33 (1H, s, imidazo[1,2-*a*]pyridine–CH), 8.51 (1H, d, *J* = 6.75 Hz, imidazo[1,2-*a*]pyridine–CH), 10.44 (1H, s, NH), 10.55 (1H, s, NH). ^13^C NMR (75 MHz, DMSO-*d*_6_): 38.86 (C), 109.07 (C), 112.70 (C), 116.90 (C), 119.35 (2C), 119.74 (2C), 125.39 (C), 125.82 (C), 126.57 (2C), 127.29 (C), 129.41 (2C), 129.67 (C), 138.82 (C), 139.67 (C), 144.51 (C), 145.19 (C), 153.30 (C), 165.14 (C), 166.05 (C). Anal. Calcd for C_23_H_17_N_6_OS_2_Cl: C, 56.03; H, 3.48; N, 17.05. Found: C, 56.17; H, 3.48; N, 17.10. HRMS (*m*/*z*): [M+2H]^+2^/2 calcd for C_23_H_17_ClN_6_OS_2_, 247.0370; found, 247.0359.

##### 4.1.8.7 2-[(5-(3-Chlorophenyl)amino-1,3,4-thiadiazol-2-yl)thio]-*N*-[4-(imidazo[1,2-*a*]pyridin-2-yl) phenyl]acetamide (**8g**)

Last step yield: 67%, mp = 199.2 °C. ^1^H NMR (300 MHz, DMSO-*d*_6_): δ = 3.55 (2H, s, CH_2_), 6.88 (1H, t, *J* = 6.75 Hz, imidazo[1,2-*a*]pyridine–CH), 6.97–7.01 (1H, m, benzene–CH), 7.23 (1H, t, *J* = 6.78 Hz, imidazo[1,2-*a*]pyridine–CH), 7.32–7.34 (3H, m, benzene–CH), 7.55 (1H, d, *J* = 9.03 Hz, imidazo[1,2-*a*]pyridine–CH), 7.67 (2H, d, *J* = 8.73 Hz, 1,4-disubstituted benzene–CH), 7.88–7.90 (2H, m, 1,4-disubstituted benzene–CH), 8.32 (1H, s, imidazo[1,2-*a*]pyridine–CH), 8.50 (1H, d, *J* = 6.72 Hz, imidazo[1,2-*a*]pyridine–CH), 10.20 (1H, s, NH), 10.28 (1H, s, NH). ^13^C NMR (75 MHz, DMSO-*d*_6_): 37.82 (C), 108.37 (C), 109.03 (C), 112.68 (C), 115.69 (C), 115.90 (C), 116.72 (C), 116.96 (C), 119.82 (2C), 121.22 (C), 124.53 (C), 125.31 (C), 126.52 (2C), 127.28 (C), 128.29 (C), 131.05 (C), 139.04 (C), 142.76 (C), 156.09 (C), 162.85 (C), 168.08 (C). Anal. Calcd For C_23_H_17_N_6_OS_2_Cl: C, 56.03; H, 3.48; N, 17.05. Found: C, 56.20; H, 3.47; N, 17.08. HRMS (*m*/*z*): [M + 2H]^+2^/2 calcd for C_23_H_17_ClN_6_OS_2_, 247.0370; found, 247.0368.

##### 4.1.8.8 2-[(5-(3-Methoxyphenyl)amino-1,3,4-thiadiazol-2-yl)thio]-*N*-[4-(imidazo[1,2-*a*]pyridin-2-yl) phenyl]acetamide (**8h**)

Last step yield: 70%, mp = 138.8 °C. ^1^H NMR (300 MHz, DMSO-*d*_6_): δ = 3.73 (3H, s, OCH_3_), 4.16 (2H, s, CH_2_), 6.57 (1H, dd, *J*_1_ = 1.89 Hz, *J*_2_ = 7.71 Hz, benzene–CH), 6.90 (1H, t, *J* = 5.85 Hz, imidazo[1,2-*a*]pyridine–CH), 7.04 (1H, dd, *J*_1_ = 1.35 Hz, *J*_2_ = 7.53 Hz, benzene–CH), 7.20–7.28 (3H, m, imidazo[1,2-*a*]pyridine–CH, benzene–CH), 7.57 (1H, d, *J* = 9.12 Hz, imidazo[1,2-*a*]pyridine–CH), 7.67 (2H, d, *J* = 8.70 Hz, 1,4-disubstituted benzene–CH), 7.92 (2H, d, *J* = 8.64 Hz, 1,4-disubstituted benzene–CH), 8.34 (1H, s, imidazo[1,2-*a*]pyridine–CH), 8.52 (1H, d, *J* = 6.72 Hz, imidazo[1,2-*a*]pyridine–CH), 10.42 (1H, s, NH), 10.44 (1H, s, NH). ^13^C NMR (75 MHz, DMSO-*d*_6_): 38.86 (C), 55.51 (C),103.46 (C), 103.80 (C), 107.86 (C), 109.11 (C), 110.26 (C), 112.83 (C), 115.45 (C), 116.80 (C), 119.77 (2C), 125.60 (C), 126.59 (2C), 127.35 (C), 129.50 (C), 130.40 (C), 138.86 (C), 141.92 (C), 145.08 (C), 160.38 (C), 165.46 (C), 166.10 (C). Anal. Calcd For C_24_H_20_N_6_O_2_S_2_: C, 58.99; H, 4.13; N, 17.20. Found: C, 59.11; H, 4.11; N, 17.16. HRMS (*m*/*z*): [M+2H]^+2^/2 calcd for C_24_H_20_N_6_O_2_S_2_, 245.0617; found, 245.0603.

##### 4.1.8.9 2-[(5-(2-Methoxyphenyl)amino-1,3,4-thiadiazol-2-yl)thio]-*N*-[4-(imidazo[1,2-*a*]pyridin-2-yl) phenyl]acetamide (**8i**)

Last step yield: 73%, mp = 104.9 °C. ^1^H NMR (300 MHz, DMSO-*d*_6_): δ = 3.84 (3H, s, OCH_3_), 4.13 (2H, s, CH_2_), 6.86 (1H, dd, *J*_1_ = 1.02 Hz, *J*_2_ = 6.72 Hz, benzene–CH), 6.91 (1H, dd, *J*_1_ = 1.62 Hz, *J*_2_ = 8.67 Hz, imidazo[1,2-*a*]pyridine–CH), 6.95–6.97 (1H, m, benzene–CH), 7.01–7.02 (1H, m, benzene–CH), 7.20–7.26 (1H, m, imidazo[1,2-*a*]pyridine–CH), 7.55 (1H, d, *J* = 9.00 Hz, imidazo[1,2-*a*]pyridine–CH), 7.66 (2H, d, *J* = 8.70 Hz, 1,4-disubstituted benzene–CH), 7.92 (2H, d, *J* = 8.67 Hz, 1,4-disubstituted benzene–CH), 8.23 (1H, dd, *J*_1_ = 1.53 Hz, *J*_2_ = 7.65 Hz, benzene–CH), 8.33 (1H, s, imidazo[1,2-*a*]pyridine–CH), 8.50 (1H, d, *J* = 6.75 Hz, imidazo[1,2-*a*]pyridine–CH), 9.80 (1H, s, NH), 10.42 (1H, s, NH). ^13^C NMR (75 MHz, DMSO-*d*_6_): 38.83 (C), 56.13 (C), 109.05 (C), 111.44 (C), 112.68 (C), 116.92 (C), 119.22 (C), 119.77 (2C), 120.18 (C), 121.06 (C), 123.18 (C), 125.34 (C), 126.54 (2C), 127.27 (C), 129.69 (C), 129.82 (C), 138.82 (C), 144.57 (C), 145.22 (C), 148.57 (C), 165.93 (C), 166.12 (C). Anal. Calcd For C_24_H_20_N_6_O_2_S_2_: C, 58.99; H, 4.13; N, 17.20. Found: C, 59.20; H, 4.12; N, 17.24. HRMS (*m*/*z*): [M + 2H]^+2^/2 calcd for C_24_H_20_N_6_O_2_S_2_, 245.0617; found, 245.0612.

##### 4.1.7.10 2-[(5-(2-Chlorophenyl)amino-1,3,4-thiadiazol-2-yl)thio]-*N*-[4-(imidazo[1,2-*a*]pyridin-2-yl) phenyl]acetamide (**8j**)

Last step yield: 72%, mp = 129.0 °C. ^1^H NMR (300 MHz, DMSO-*d*_6_): δ = 4.15 (2H, s, CH_2_), 6.89 (1H, t, *J* = 6.54 Hz, imidazo[1,2-*a*]pyridine–CH), 7.08 (1H, t, *J* = 7.53 Hz, benzene–CH), 7.21–7.26 (1H, m, imidazo[1,2-*a*]pyridine–CH), 7.34 (1H, t, *J* = 7.41 Hz, benzene–CH), 7.48 (1H, dd, *J*_1_ = 1.29 Hz, *J*_2_ = 8.01 Hz, benzene–CH), 7.56 (1H, d, *J* = 8.97 Hz, imidazo[1,2-*a*]pyridine–CH), 7.67 (2H, d, *J* = 8.76 Hz, 1,4-disubstituted benzene–CH), 7.92 (2H, d, *J* = 8.67 Hz, 1,4-disubstituted benzene–CH), 8.23 (1H, d, *J* = 8.19 Hz, benzene–CH), 8.34 (1H, s, imidazo[1,2-*a*]pyridine–CH), 8.52 (1H, d, *J* = 6.75 Hz, imidazo[1,2-*a*]pyridine–CH), 9.88 (1H, s, NH), 10.43 (1H, s, NH). ^13^C NMR (75 MHz, DMSO-*d*_6_): 38.79 (C), 109.13 (C), 109.94 (C), 112.74 (C), 113.63 (C), 115.79 (C), 116.91 (C), 117.21 (C), 119.76 (2C), 121.98 (C), 124.62 (C), 125.43 (C), 126.57 (2C), 127.29 (C), 128.39 (C), 129.62 (C), 130.15 (C), 131.30 (C), 138.83 (C), 144.47 (C), 166.06 (C). Anal. Calcd For C_23_H_17_N_6_OS_2_Cl: C, 56.03; H, 3.48; N, 17.05. Found: C, 55.90; H, 3.47; N, 17.09. HRMS (*m*/*z*): [M + 2H]^+2^/2 calcd for C_23_H_17_ClN_6_OS_2_, 247.0370; found, 247.0362.

##### 4.1.8.11 2-[(5-(4-Methoxyphenyl)amino-1,3,4-thiadiazol-2-yl)thio]-*N*-[4-(imidazo[1,2-*a*]pyridin-2-yl) phenyl]acetamide (**8k**)

Last step yield: 70%, mp = 216.5 °C. ^1^H NMR (300 MHz, DMSO-*d*_6_): 3.75 (3H, s, OCH_3_), 4.15 (2H, s, CH_2_), 6.90–6.96 (2H, m, imidazo[1,2-*a*]pyridine–CH, benzene–CH), 7.13–7.18 (1H, s, benzene–CH), 7.24–7.29 (1H, m, imidazo[1,2-*a*]pyridine–CH), 7.45–7.52 (2H, m, benzene–CH), 7.58–7.61 (1H, m, imidazo[1,2-*a*]pyridine–CH), 7.65–7.71 (2H, m, benzene–CH), 7.93–7.97 (2H, m, benzene–CH), 8.37 (1H, s, imidazo[1,2-*a*]pyridine–CH), 8.54–8.56 (1H, m, imidazo[1,2-*a*]pyridine–CH), 10.24 (1H, s, NH), 10.44 (1H, s, NH). ^13^C NMR (75 MHz, DMSO-*d*_6_): 38.72 (C), 54.71 (C), 107.90 (C), 109.31 (C), 110.46 (C), 113.66 (C), 114.04 (C), 115.85 (C), 117.71 (C), 118.66 (2C), 120.83 (2C), 125.50 (C), 126.16 (2C), 127.63 (2C), 128.73 (C), 129.23 (C), 134.24 (C), 138.83 (C), 138.95 (C), 166.28 (C). Anal. Calcd For C_24_H_20_N_6_O_2_S_2_: C, 58.99; H, 4.13; N, 17.20. Found: C, 59.17; H, 4.12; N, 17.22. HRMS (*m*/*z*): [M + 2H]^+2^/2 calcd for C_24_H_20_N_6_O_2_S_2_, 245.0617; found, 245.0608.

### 4.2 Biological Evaluation

#### 4.2.1 AR Activity

The activity was assessed using methods that were modified from previously established protocols.^48,49^ The inhibitory activity of AR was assessed by measuring the reduction in absorbance at 340 nm due to the consumption of NADPH. The reaction mixture for AR activity comprised 0.8 M sodium phosphate buffer (pH 5.5), NADPH (0.11 mM), dl-glyceraldehyde (4.7 mM), and enzyme solution.

#### 4.2.2 α-Glycosidase Activity

The inhibitory activity of α-GLY was rigorously evaluated using *para*-nitrophenyl-α-d-glucopyranoside as the substrate, following the detailed experimental methodology described by Tao et al.,^41^ with acarbose as the control inhibitor. In this analysis, a unit of α-GLY activity is defined as the amount of enzyme required to catalyze the hydrolysis of 1 mol of substrate per minute at a standard pH of 7.4.

#### 4.2.3 α-Amylase Activity

The α-AMY inhibitory activity was determined using the method described by Xiao et al.,^42^ with acarbose as the control inhibitor. The assay involved preparing a substrate solution of starch in NaOH, adjusting the pH with HCl, and diluting the solution to volume. Enzyme and sample solutions were preincubated, mixed with the substrate, and incubated again. The reaction was stopped with HCl, and the absorbance was measured at 580 nm.

##### 4.2.3.1 In Vitro Inhibition Studies

The inhibition effects of the novel imidazo[1,2-*a*]pyridine-based 1,3,4-thiadiazole derivatives were determined with different inhibitor concentrations (at least five) against AR, α-GLY, and α-AMY. The IC_50_ of the derivatives was calculated from Activity (%) – [inhibitor] graphs for derivatives. The inhibition types and *K*_I_ values were found by Lineweaver and Burk’s curves.^50,51^

### 4.3 Molecular Docking Study

In this study, the molecular docking analyses were conducted using the latest iteration of the Schrödinger Small-Molecule Drug Discovery Suite for Mac, version 2024-2. Protein structures with PDB IDs 4JIR(45) and 5NN8,^46^ corresponding to AR and α-GLY, respectively, were retrieved from the RCSB Protein Data Bank. These structures were preprocessed for docking using the Protein Preparation Wizard within the Small-Molecule Drug Discovery Suite. The initial refinement of the preprocessed protein structure involved optimizing the orientation of the sample-water molecules, followed by a restrained minimization of the cocrystallized complex using the OPLS4 force field. This procedure reoriented side chain hydroxyl groups and resolved potential steric clashes. The complex was then minimized until it achieved convergence with a heavy atom RMSD of 0.3 Å. Novel imidazo[1,2-*a*]pyridine-based 1,3,4-thiadiazoles (**8a**–**k**) were designed using ChemDraw version 21 (PerkinElmer, Inc., Waltham, MA, USA) for Mac and subsequently optimized employing the LigPrep module at pH 7.4 ± 0.5, utilizing the OPLS4 force field with Epik. The active site residues, identified by the SiteMap tool, were specified in the Receptor Grid Generation module to create the receptor grid at the Maestro interface. Docking of the ligands to AR and α-GLY was performed by using the Glide application with default parameters and the extra precision (XP) methodology. The Prime MM-GBSA method was evaluated for its efficacy in predicting relative binding affinities using the VSGB energy model and OPLS4 force field on protein–ligand complexes 4JIR and 5NN8.

#### 4.3.1 Statistical Studies

Data analysis and graphical representations were conducted using GraphPad Prism version 8 for Mac (GraphPad Software, La Jolla, California, USA). Model adequacy for enzyme inhibition was evaluated through comparison using the extra sum-of-squares F test alongside the AICc method. Outcomes are expressed as the mean ± standard error of the mean and include 95% confidence intervals. A *p*-value of less than 0.05 was considered statistically significant.

## Supporting Information Available

^1^H NMR, ^13^C NMR, and HRMS spectra for all compounds (PDF)
