# Supplementary material for: Synthesis, α-Glucosidase, α-Amylase, and Aldol Reductase Inhibitory Activity with Molecular Docking Study of Novel Imidazo[1,2-a]pyridine Derivatives
Source: ACS Omega. 2024 Oct 11;9(42):42905–14. doi: 10.1021/acsomega.4c05619 (PMC11500159; doi:10.1021/acsomega.4c05619)
Supplement: Supplementary file 1 — ao4c05619_si_001.pdf [file ao4c05619_si_001.pdf]

Synthesis, α-glucosidase, α-amylase and aldol reductase inhibitory activity with molecular docking study of novel imidazo[1,2-a]pyridine derivatives

Betül Kaya[a], Ulviye Acar Çevik[b], Bilge Çiftçi[c], Hatice Esra Duran[d], Cüneyt Türkeş[e], Mesut Işık[f], Hayrani Eren Bostancı[g*], Zafer Asım Kaplancıklı[b], Şükrü Beydemir[h]

[a]Department of Pharmaceutical Chemistry, Faculty of Pharmacy, Zonguldak Bulent Ecevit University, 67600 Zonguldak, Turkey

[b]Department of Pharmaceutical Chemistry, Faculty of Pharmacy, Anadolu University, 26470 Eskişehir, Turkey

[c]Vocational School of Health Services, Bilecik Şeyh Edebali University, 11230, Bilecik, Turkey

[d]Department of Medical Biochemistry, Faculty of Medicine, Kafkas University, Kars 36100, Turkey

[e]Department of Biochemistry, Faculty of Pharmacy, Erzincan Binali Yıldırım University, Erzincan 24002, Turkey

[f]Department of Bioengineering, Faculty of Engineering, Bilecik Şeyh Edebali University, 11230, Bilecik, Turkey

[g]Department of Biochemistry, Faculty of Pharmacy, Cumhuriyet University, 58140 Sivas, Turkey.

[h]Department of Biochemistry, Faculty of Pharmacy, Anadolu University, 26470, Eskişehir, Turkey

*Correspondence:

Corresponding Author. E-mail: erenbostanci@cumhuriyet.edu.tr

Address: Sivas Cumhuriyet University, Faculty of Pharmacy, Department of Biochemistry Sivas, Turkey.

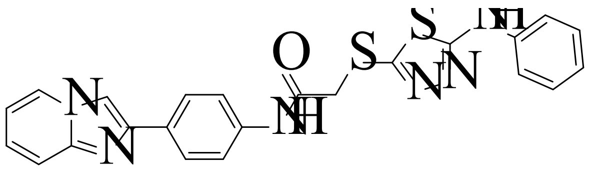

Figure S1. The chemical structure of compound **8a**

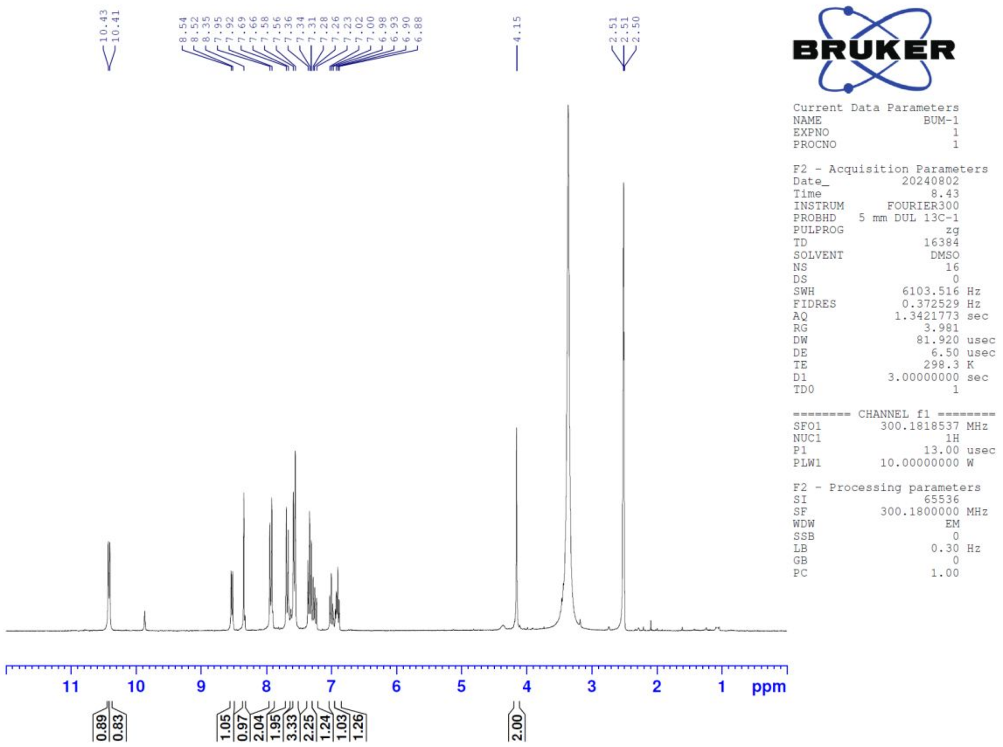

Figure S2. ^{1}H-NMR spectrum of compound **8a**

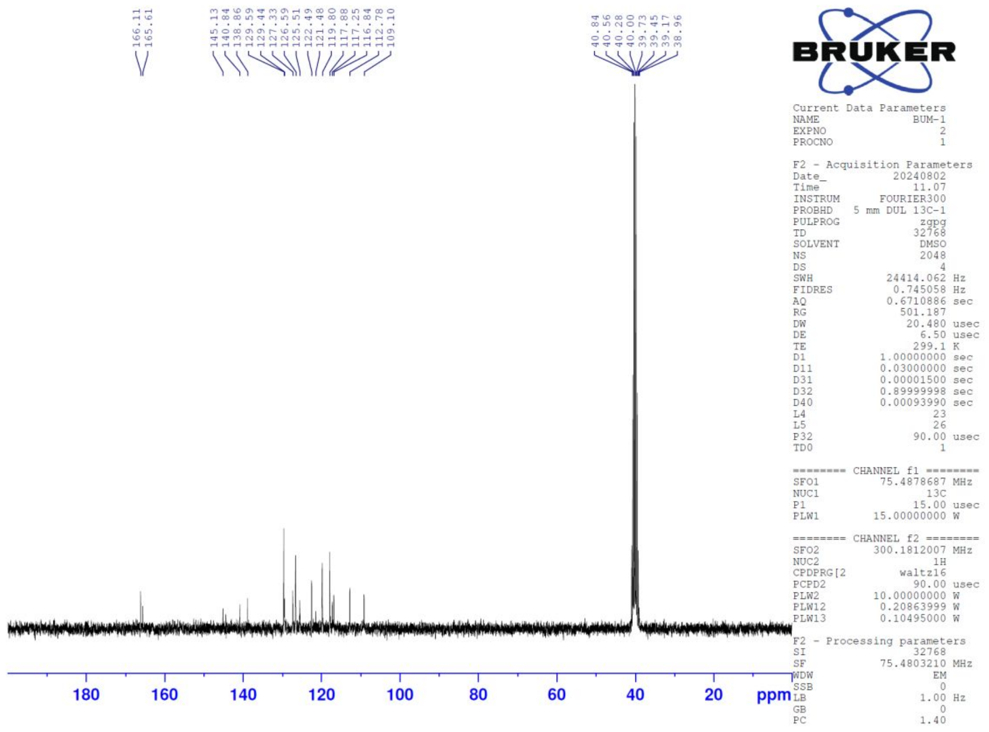

Figure S3. ^{13}C-NMR spectrum of compound **8a**

Data File: C:\LabSolutions\Data\Analiz\uac\BUM-1_224.lcd

Elmt	Val.	Min	Max	Elmt	Val.	Min	Max	Elmt	Val.	Min	Max	Elmt	Val.	Min	Max	Use Adduct
H	1	8	33	O	2	0	5	S	2	0	2	Ru	2	0	0	H
C	4	4	32	F	1	0	0	Cl	1	0	0	Pd	2	0	0	
N	3	0	6	P	3	0	0	Br	1	0	0	I	3	0	0	

Error Margin (ppm):	5	DBE Range:	0.0 - 30.0	Electron Ions:	both
HC Ratio:	unlimited	Apply N Rule:	no	Use MSn Info:	yes
Max Isotopes:	3	Isotope RI (%):	1.00	Isotope Res:	9000
MSn Iso RI (%):	10.00	MSn Logic Mode:	AND	Max Results:	50

Event#: 1 MS(E+) Ret. Time : 2.253 Scan# : 339

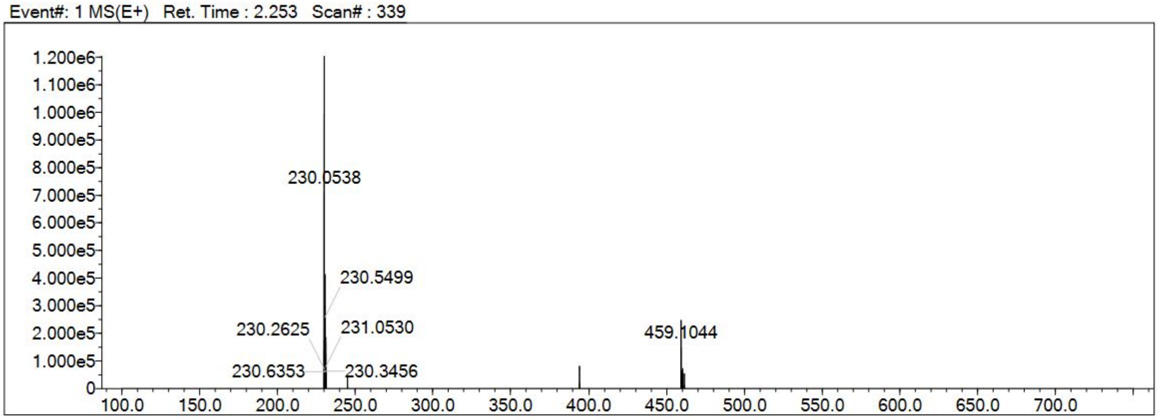

Measured region for 459.1044 m/z

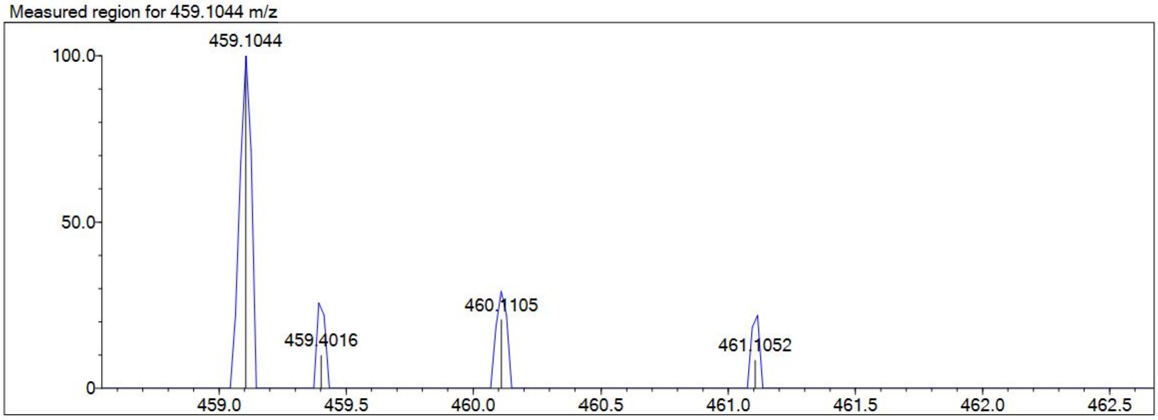

C23 H18 N6 O S2 [M+H]+ : Predicted region for 459.1056 m/z

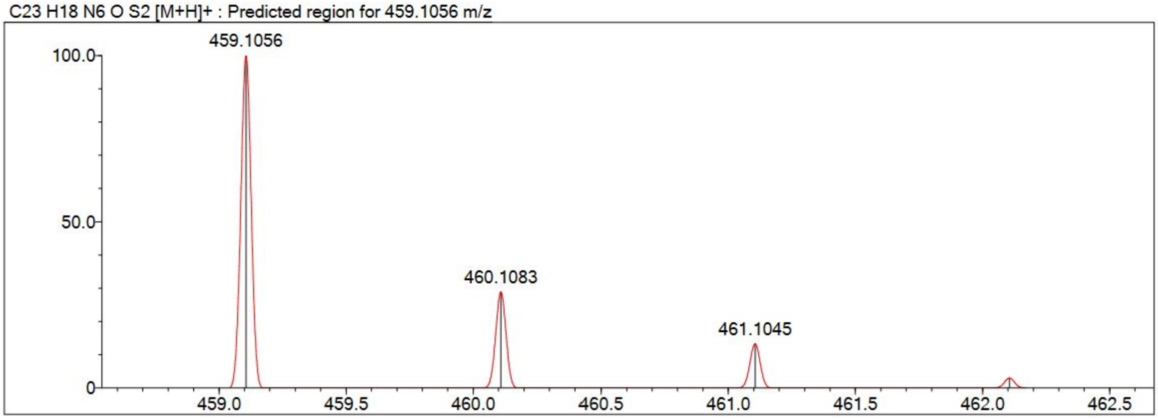

Rank	Score	Formula (M)	Ion	Meas. m/z	Pred. m/z	Df. (mDa)	Df. (ppm)	Iso	DBE
2	71.38	C23 H18 N6 O S2	[M+H]+	459.1044	459.1056	-1.2	-2.61	74.38	18.0

Figure S4. Mass spectrum of compound **8a**

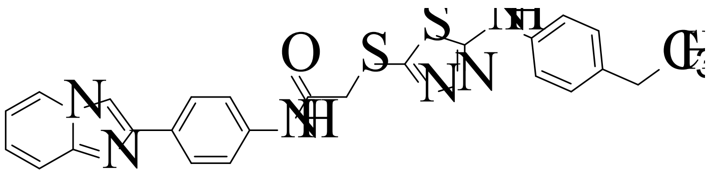

Figure S5. The chemical structure of compound **8b**

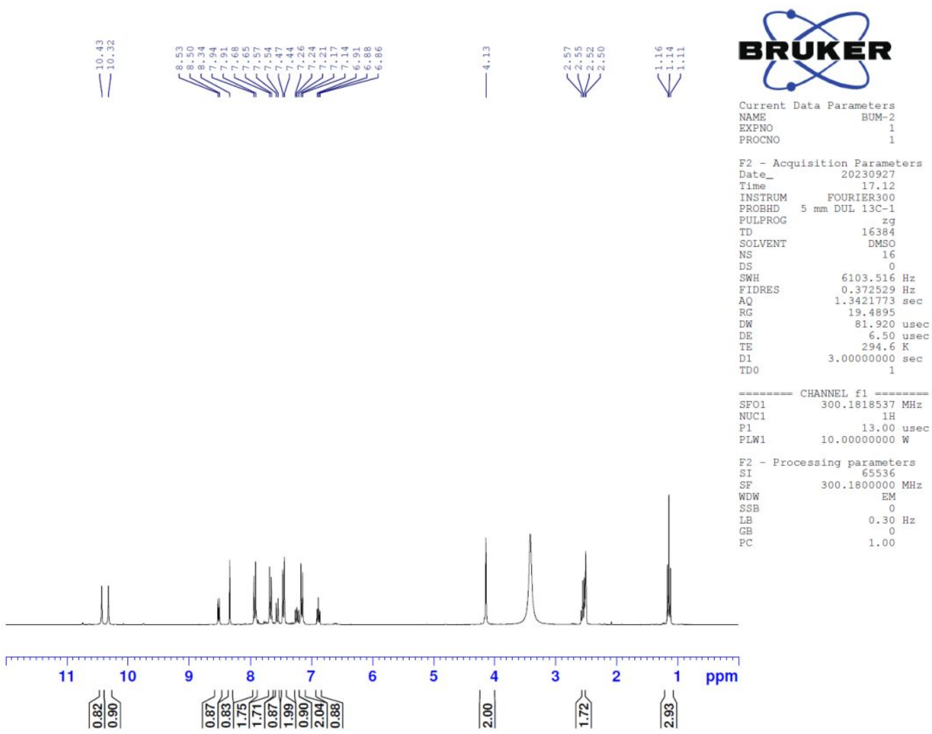

Figure S6. ^{1}H-NMR spectrum of compound **8b**

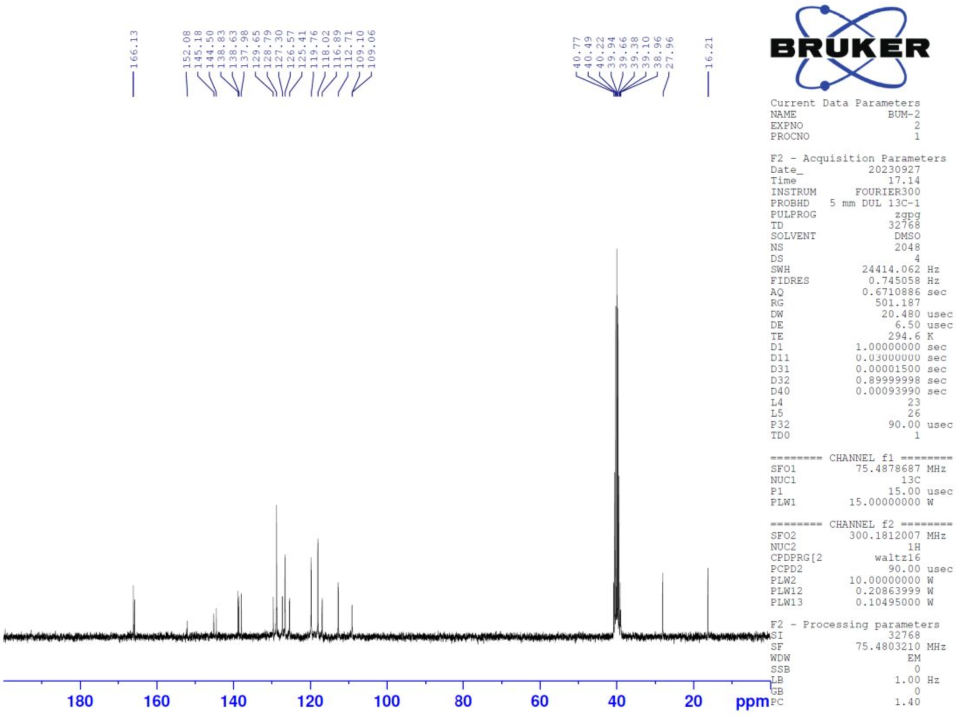

Figure S7. ^{13}C-NMR spectrum of compound **8b**

Data File: C:\LabSolutions\Data\Analiz\uac\BUM-2_225.lcd

Elmt	Val.	Min	Max	Elmt	Val.	Min	Max	Elmt	Val.	Min	Max	Elmt	Val.	Min	Max	Use Adduct
H	1	8	33	O	2	0	5	S	2	0	2	Ru	2	0	0	H
C	4	4	32	F	1	0	0	Cl	1	0	0	Pd	2	0	0	
N	3	0	6	P	3	0	0	Br	1	0	0	I	3	0	0	

Error Margin (ppm): 5
HC Ratio: unlimited
Max Isotopes: 3
MSn Iso RI (%): 10.00

DBE Range: 0.0 - 30.0
Apply N Rule: no
Isotope RI (%): 1.00
MSn Logic Mode: AND

Electron Ions: both
Use MSn Info: yes
Isotope Res: 9000
Max Results: 50

Event#: 1 MS(E+) Ret. Time : 2.400 Scan# : 361

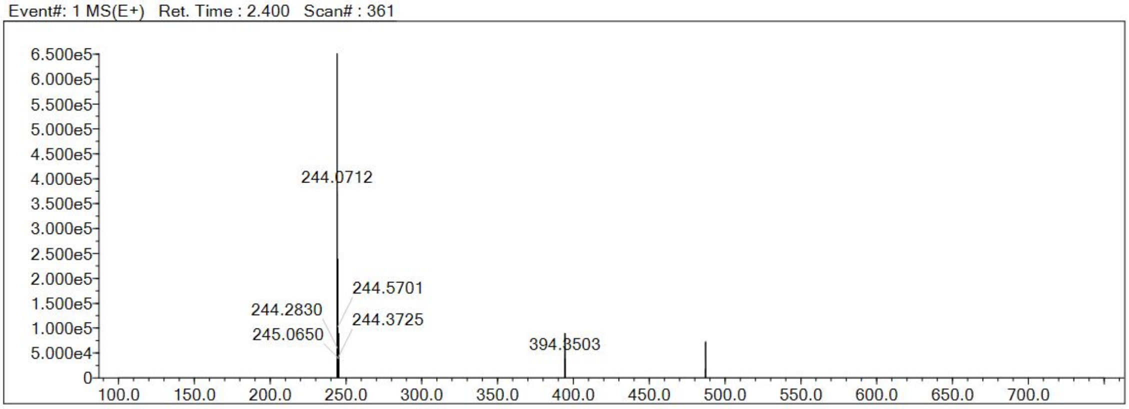

Measured region for 244.0712 m/z

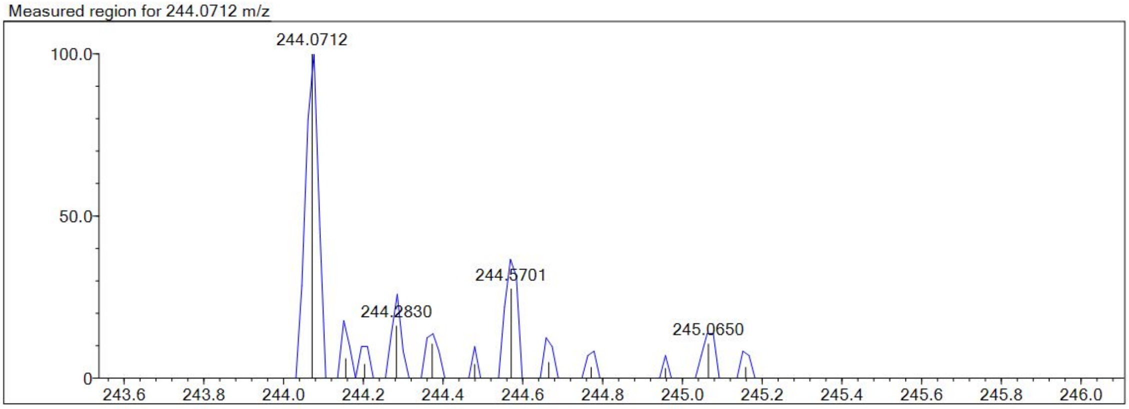

C25 H22 N6 O S2 [M+2H]2+ : Predicted region for 244.0721 m/z

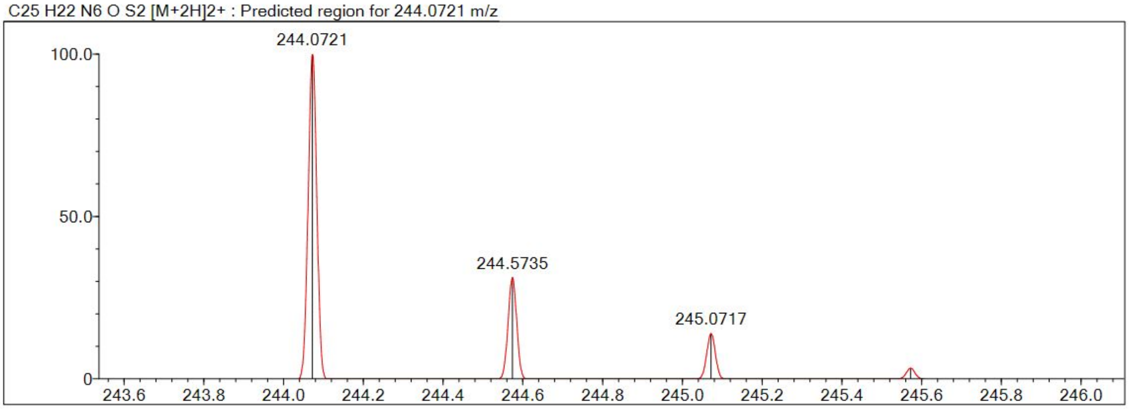

Rank	Score	Formula (M)	Ion	Meas. m/z	Pred. m/z	Df. (mDa)	Df. (ppm)	Iso	DBE
3	56.26	C25 H22 N6 O S2	[M+2H]2+	244.0712	244.0721	-0.9	-3.69	60.32	18.0

Figure S8. Mass spectrum of compound **8b**

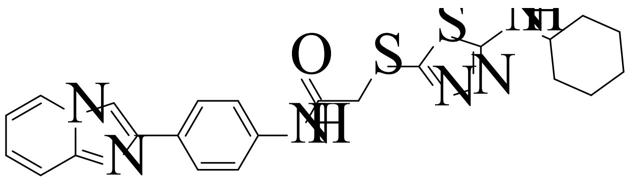

Figure S9. The chemical structure of compound **8c**

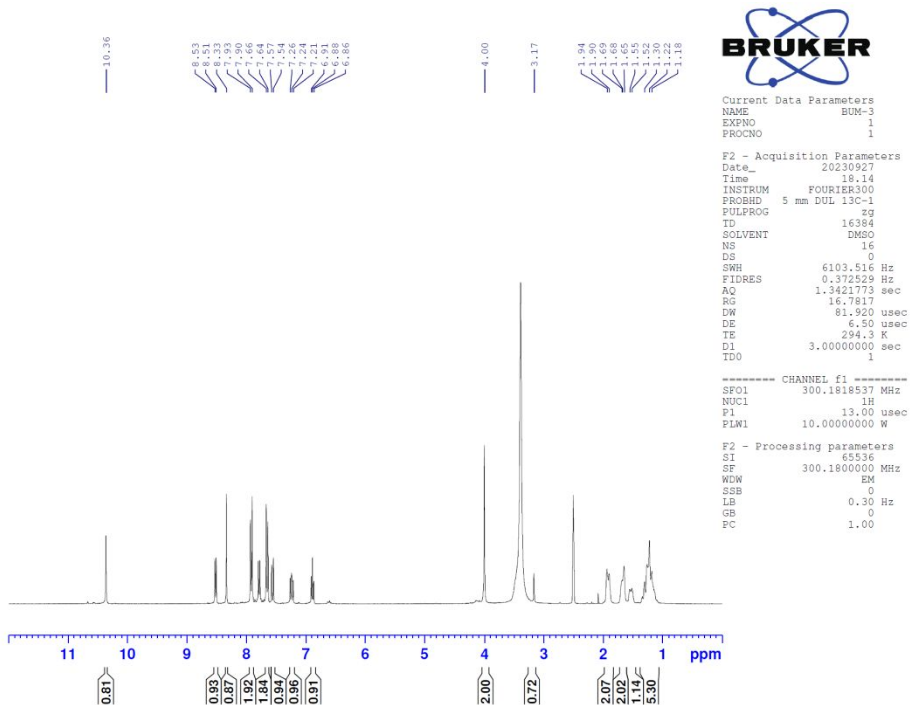

Figure S10. ^{1}H-NMR spectrum of compound **8c**

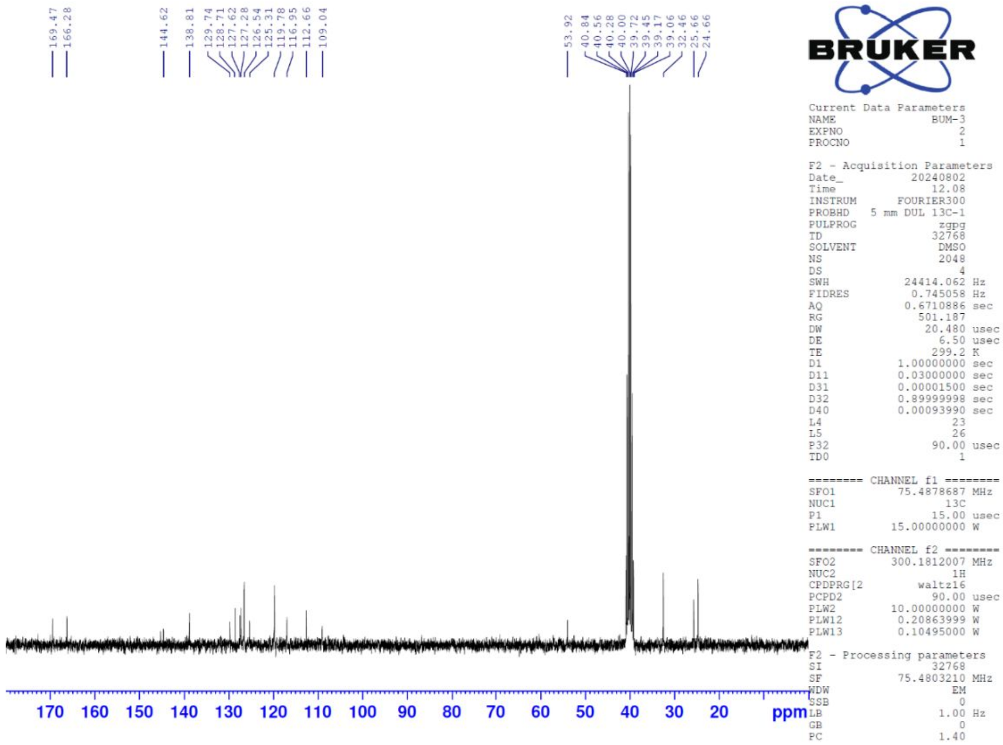

Figure S11. ^{13}C-NMR spectrum of compound **8c**

Formula Predictor Report - BUM-3_226.lcd

Data File: C:\LabSolutions\Data\Analiz\uac\BUM-3_226.lcd

Elmt	Val.	Min	Max	Elmt	Val.	Min	Max	Elmt	Val.	Min	Max	Elmt	Val.	Min	Max	Use Adduct
H	1	8	33	O	2	0	5	S	2	0	2	Ru	2	0	0	H
C	4	4	32	F	1	0	0	Cl	1	0	0	Pd	2	0	0	
N	3	6	6	P	3	0	0	Br	1	0	0	I	3	0	0	

Error Margin (ppm):	6	DBE Range:	0.0 - 30.0	Electron Ions:	both
HC Ratio:	unlimited	Apply N Rule:	no	Use MSn Info:	yes
Max Isotopes:	3	Isotope RI (%):	1.00	Isotope Res:	9000
MSn Iso RI (%):	10.00	MSn Logic Mode:	AND	Max Results:	50

Event#: 1 MS(E+) Ret. Time : 2.307 Scan# : 347

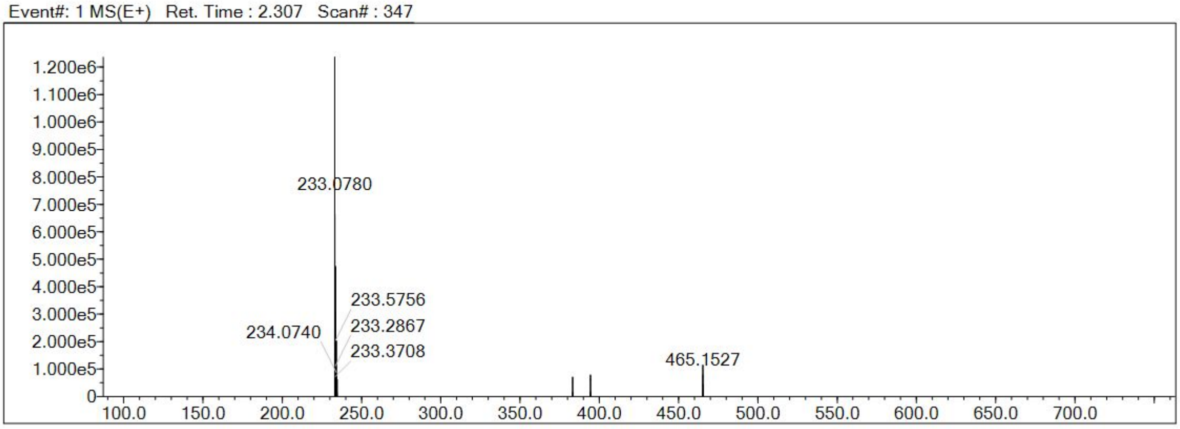

Measured region for 465.1527 m/z

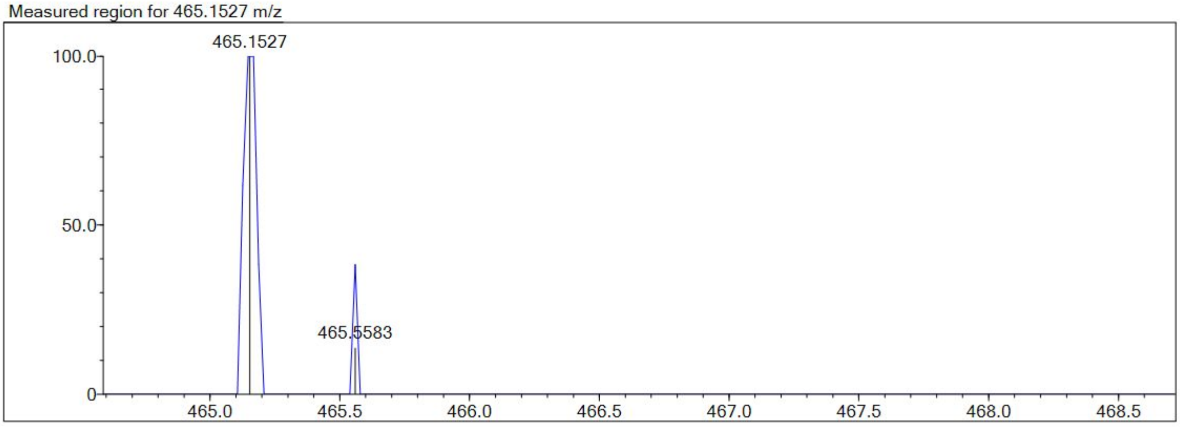

C23 H24 N6 O S2 [M+H]+ : Predicted region for 465.1526 m/z

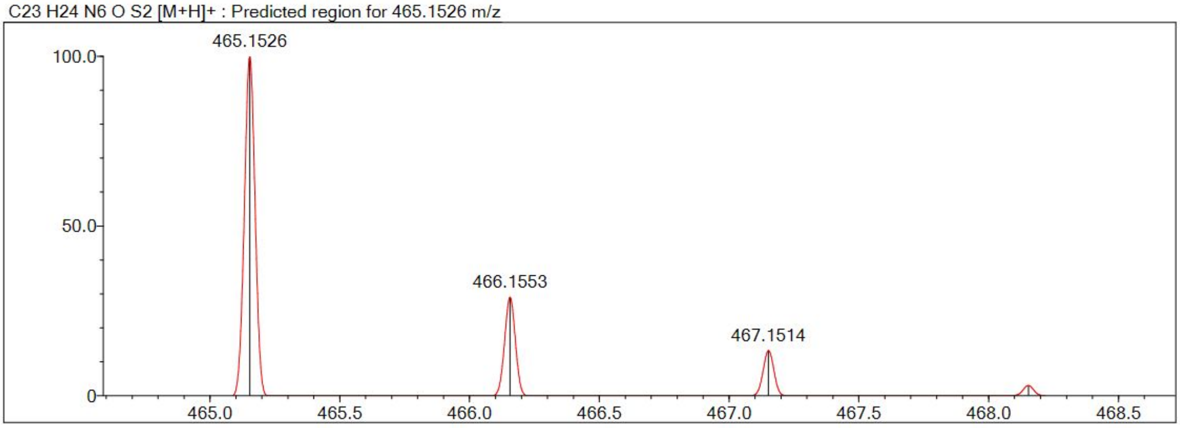

Rank	Score	Formula (M)	Ion	Meas. m/z	Pred. m/z	Df. (mDa)	Df. (ppm)	Iso	DBE
1	0.00	C23 H24 N6 O S2	[M+H]+	465.1527	465.1526	0.1	0.21	0.00	15.0

Figure S12. Mass spectrum of compound **8c**

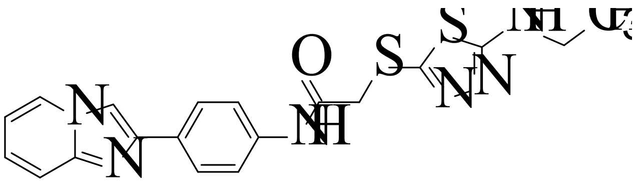

Figure S13. The chemical structure of compound **8d**

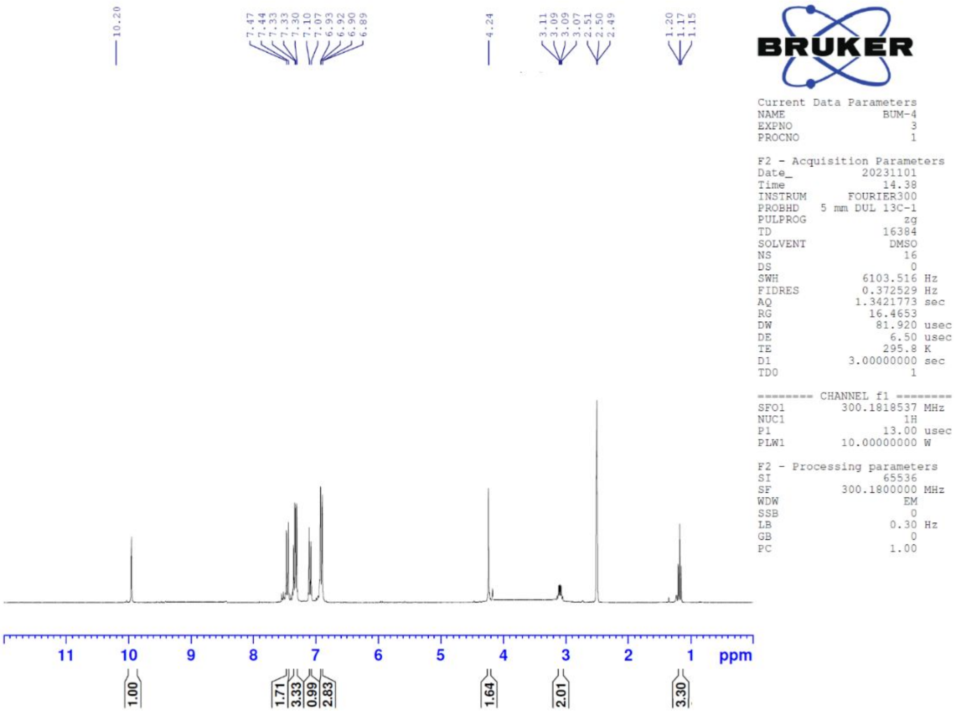

Figure S14. ^{1}H-NMR spectrum of compound **8d**

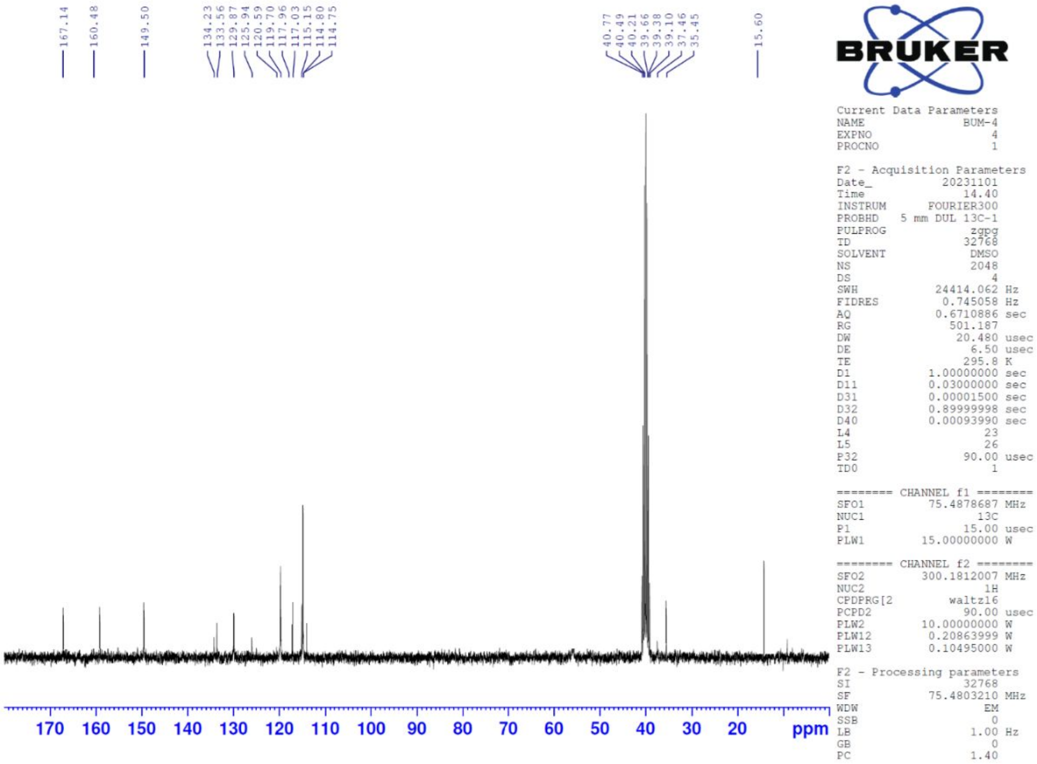

Figure S15. ^{13}C-NMR spectrum of compound **8d**

Data File: C:\LabSolutions\Data\Analiz\uac\BUM-4_227.lcd

Elmt	Val.	Min	Max	Elmt	Val.	Min	Max	Elmt	Val.	Min	Max	Elmt	Val.	Min	Max	Use Adduct
H	1	8	33	O	2	0	5	S	2	0	2	Ru	2	0	0	H
C	4	4	32	F	1	0	0	Cl	1	0	0	Pd	2	0	0	
N	3	0	6	P	3	0	0	Br	1	0	0	I	3	0	0	

Error Margin (ppm): 10
HC Ratio: unlimited
Max Isotopes: 3
MSn Iso RI (%): 10.00

DBE Range: 0.0 - 30.0
Apply N Rule: no
Isotope RI (%): 1.00
MSn Logic Mode: AND

Electron Ions: both
Use MSn Info: yes
Isotope Res: 9000
Max Results: 50

Event#: 1 MS(E+) Ret. Time : 2.387 Scan# : 359

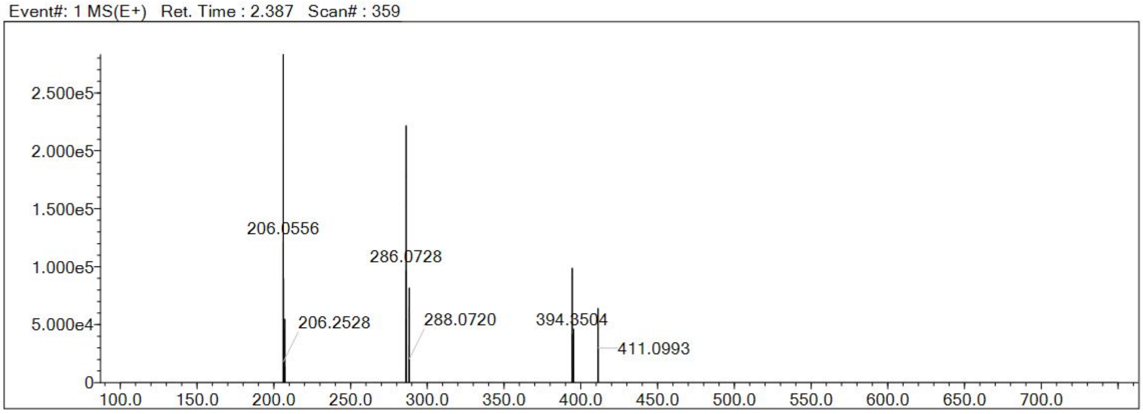

Measured region for 206.0556 m/z

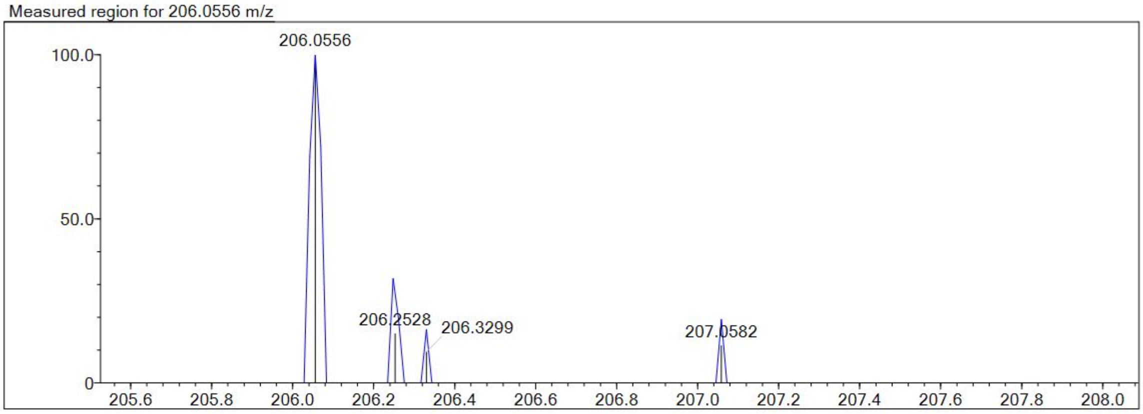

C19 H18 N6 O S2 [M+2H]2+ : Predicted region for 206.0565 m/z

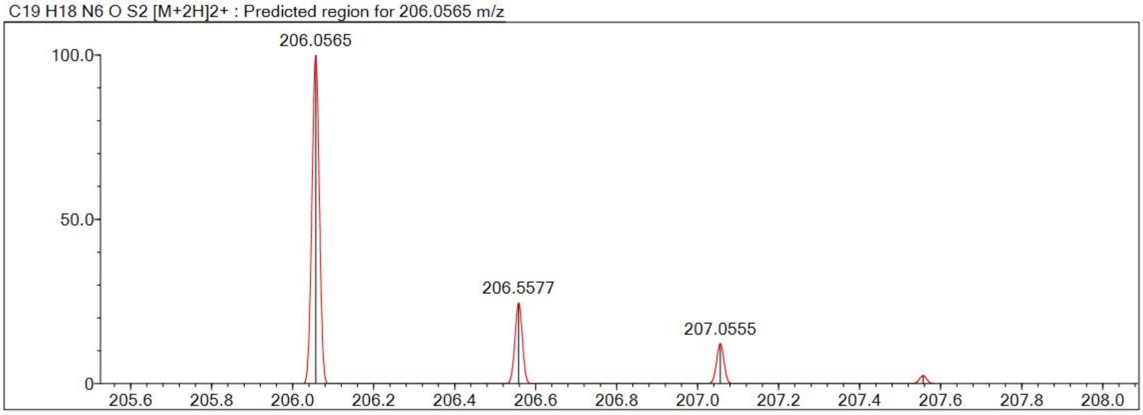

Rank	Score	Formula (M)	Ion	Meas. m/z	Pred. m/z	Df. (mDa)	Df. (ppm)	Iso	DBE
4	67.93	C19 H18 N6 O S2	[M+2H]2+	206.0556	206.0565	-0.9	-4.37	74.18	14.0

Figure S16. Mass spectrum of compound **8d**

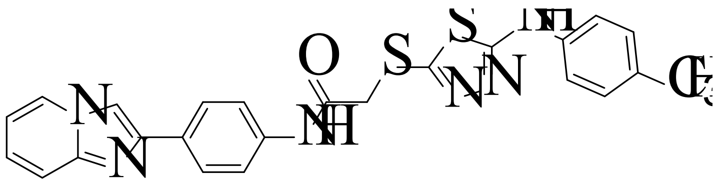

Figure S17. The chemical structure of compound **8e**

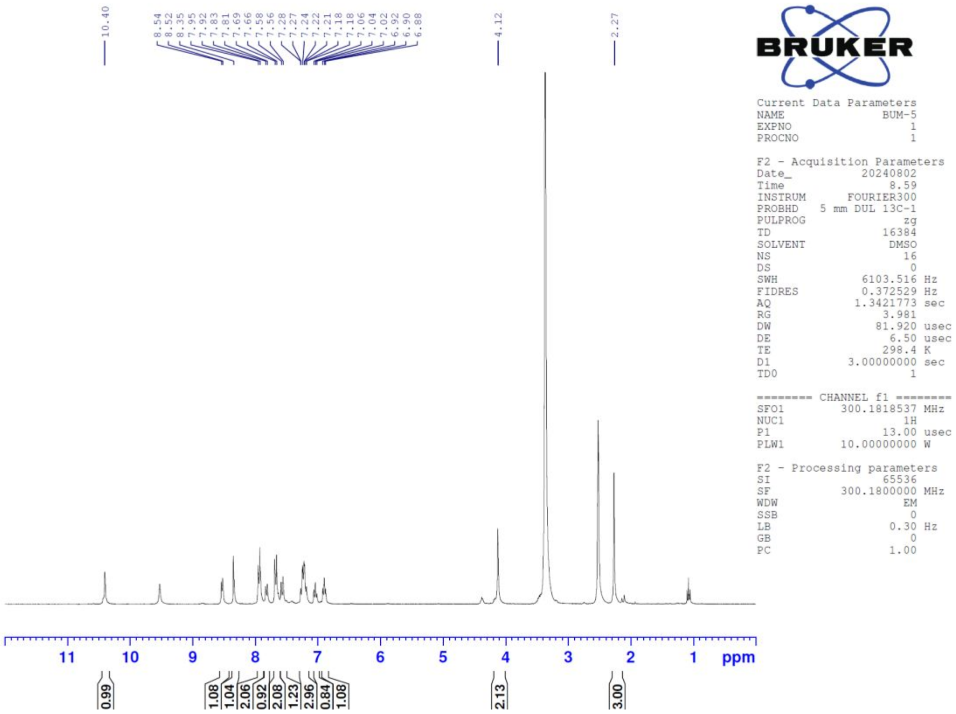

Figure S18. ^{1}H-NMR spectrum of compound **8e**

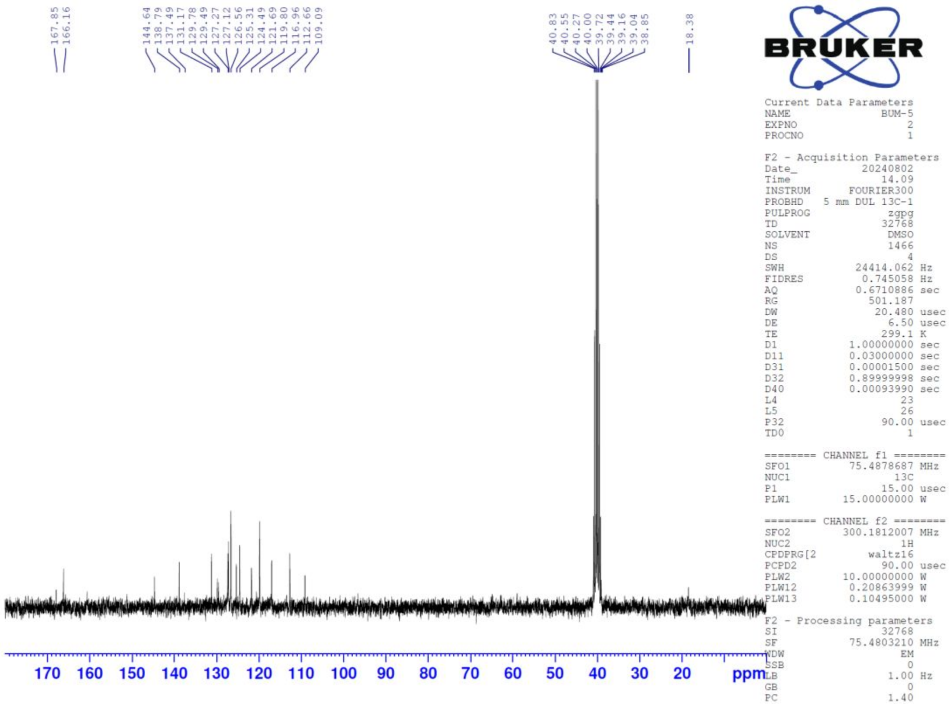

Figure S19. ^{13}C-NMR spectrum of compound **8e**

Formula Predictor Report - BUM-5_228.lcd

Data File: C:\LabSolutions\Data\Analiz\uac\BUM-5_228.lcd

Elmt	Val.	Min	Max	Elmt	Val.	Min	Max	Elmt	Val.	Min	Max	Elmt	Val.	Min	Max	Use Adduct
H	1	8	33	O	2	0	5	S	2	0	2	Ru	2	0	0	H
C	4	4	32	F	1	0	0	Cl	1	0	0	Pd	2	0	0	
N	3	6	6	P	3	0	0	Br	1	0	0	I	3	0	0	

Error Margin (ppm):	6	DBE Range:	0.0 - 30.0	Electron Ions:	both
HC Ratio:	unlimited	Apply N Rule:	no	Use MSn Info:	yes
Max Isotopes:	3	Isotope RI (%):	1.00	Isotope Res:	9000
MSn Iso RI (%):	10.00	MSn Logic Mode:	AND	Max Results:	50

Event#: 1 MS(E+) Ret. Time : 2.440 Scan# : 367

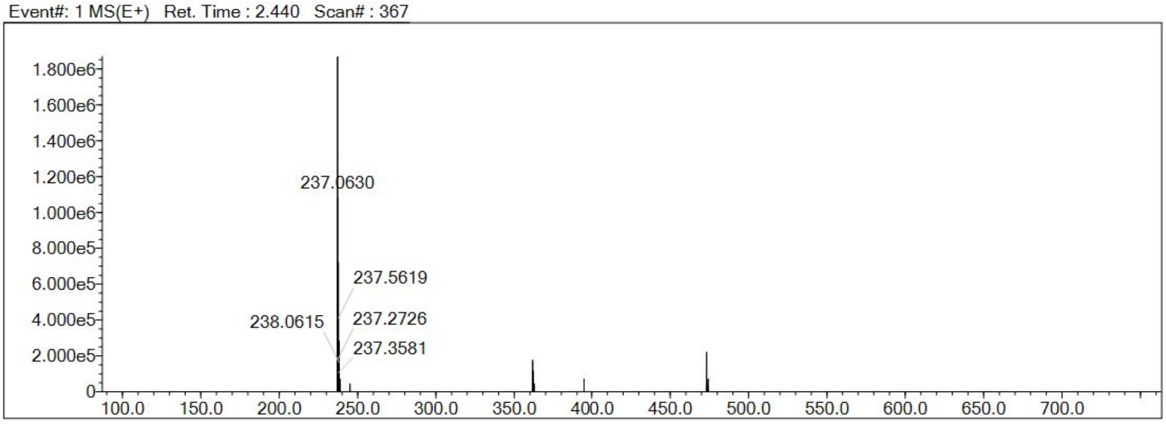

Measured region for 237.0630 m/z

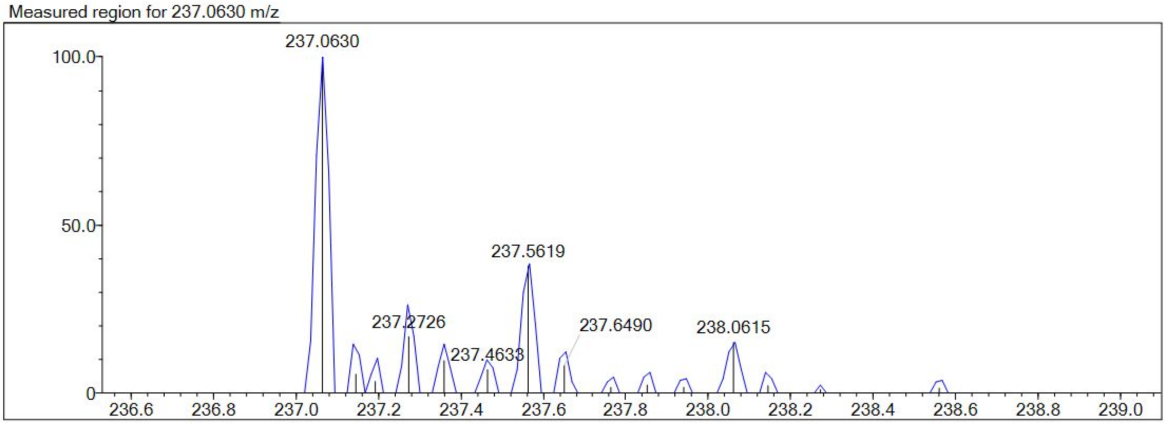

C24 H20 N6 O S2 [M+2H]2+ : Predicted region for 237.0643 m/z

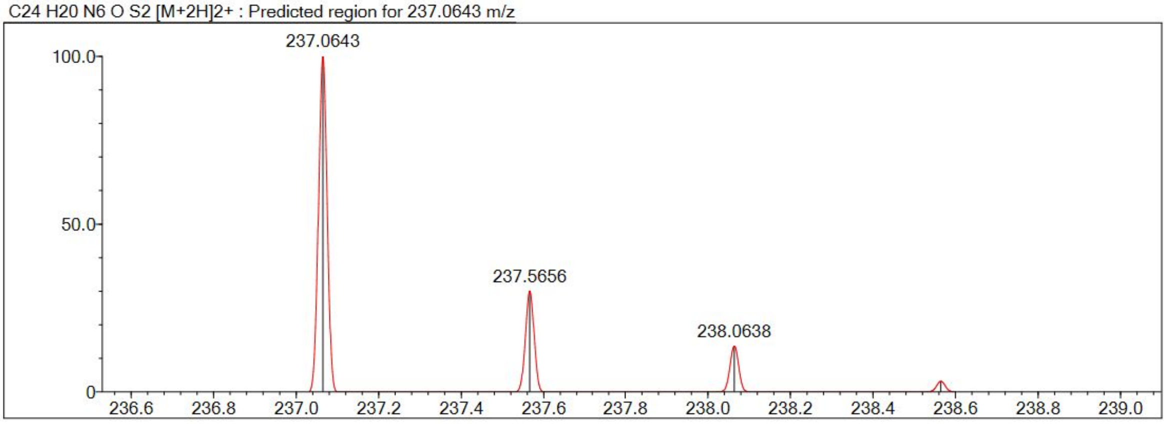

Rank	Score	Formula (M)	Ion	Meas. m/z	Pred. m/z	Df. (mDa)	Df. (ppm)	Iso	DBE
2	60.46	C24 H20 N6 O S2	[M+2H]2+	237.0630	237.0643	-1.3	-5.48	70.96	18.0

Figure S20. Mass spectrum of compound **8e**

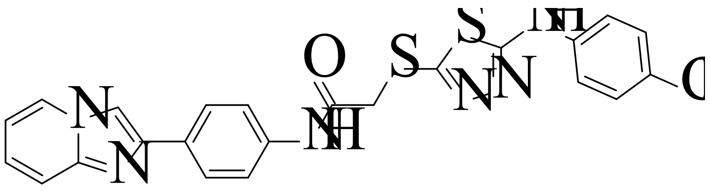

Figure S21. The chemical structure of compound **8f**

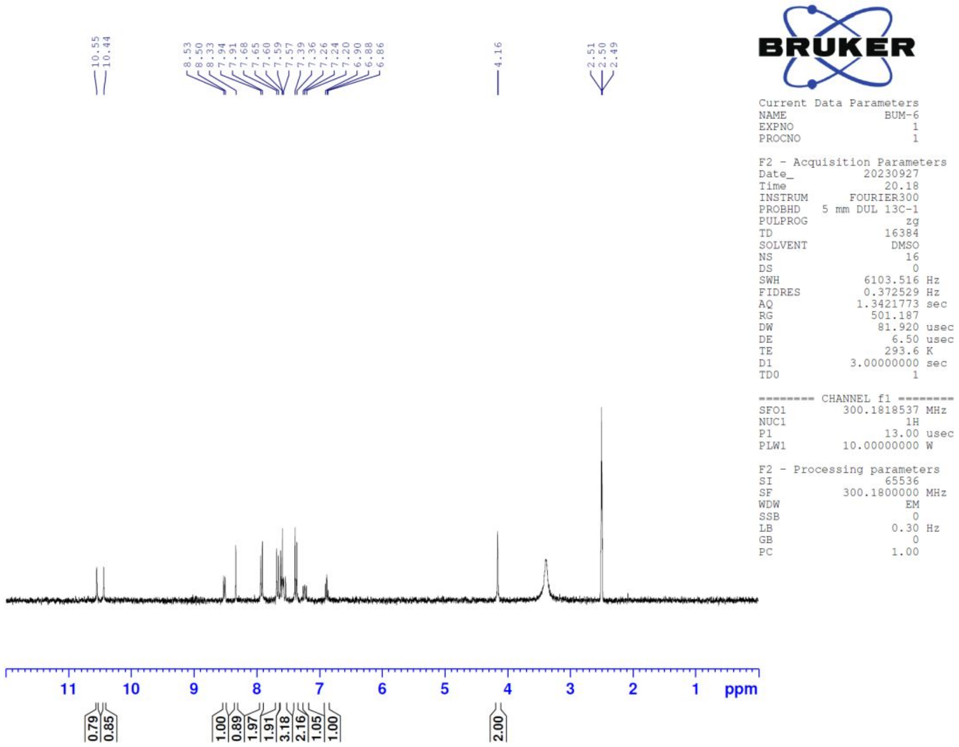

Figure S22. ^{1}H-NMR spectrum of compound **8f**

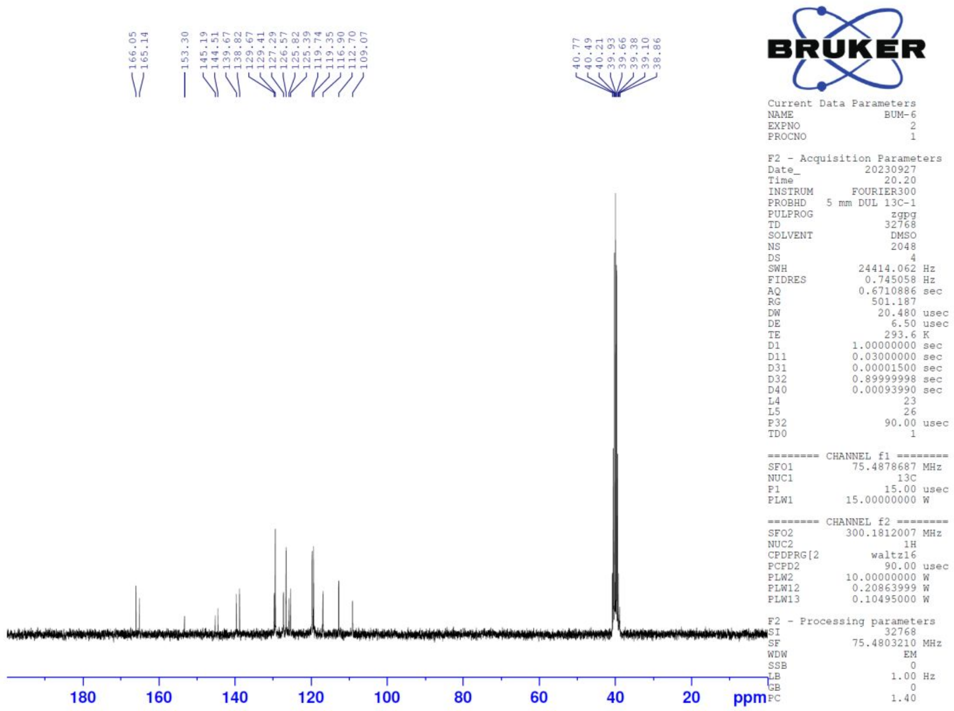

Figure S23. ^{13}C-NMR spectrum of compound **8f**

Data File: C:\LabSolutions\Data\Analiz\uac\BUM-6_229.lcd

Elmt	Val.	Min	Max	Elmt	Val.	Min	Max	Elmt	Val.	Min	Max	Elmt	Val.	Min	Max	Use Adduct
H	1	8	33	O	2	0	5	S	2	0	2	Ru	2	0	0	H
C	4	4	32	F	1	0	0	Cl	1	1	1	Pd	2	0	0	
N	3	6	6	P	3	0	0	Br	1	0	0	I	3	0	0	

Error Margin (ppm):	6	DBE Range:	0.0 - 30.0	Electron Ions:	both
HC Ratio:	unlimited	Apply N Rule:	no	Use MSn Info:	yes
Max Isotopes:	3	Isotope RI (%):	1.00	Isotope Res:	9000
MSn Iso RI (%):	10.00	MSn Logic Mode:	AND	Max Results:	50

Event#: 1 MS(E+) Ret. Time : 2.267 Scan# : 341

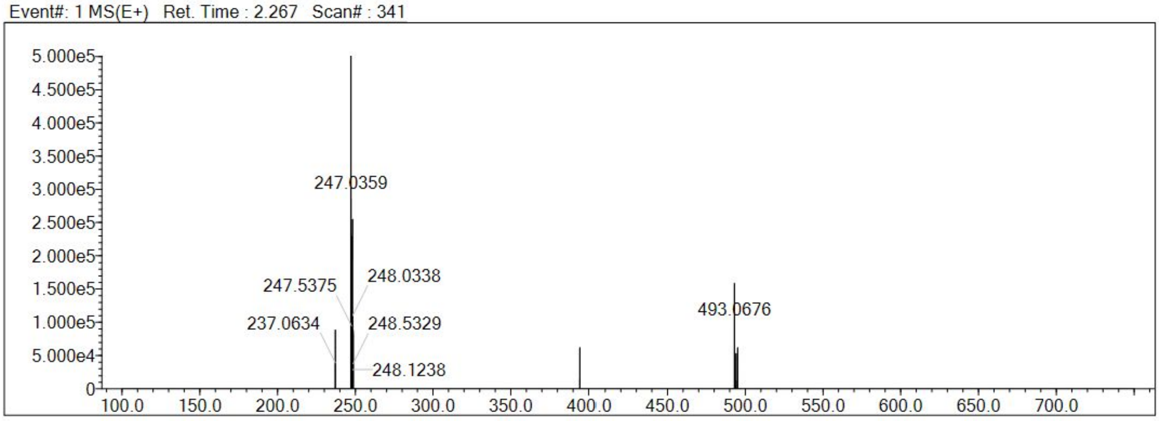

Measured region for 247.0359 m/z

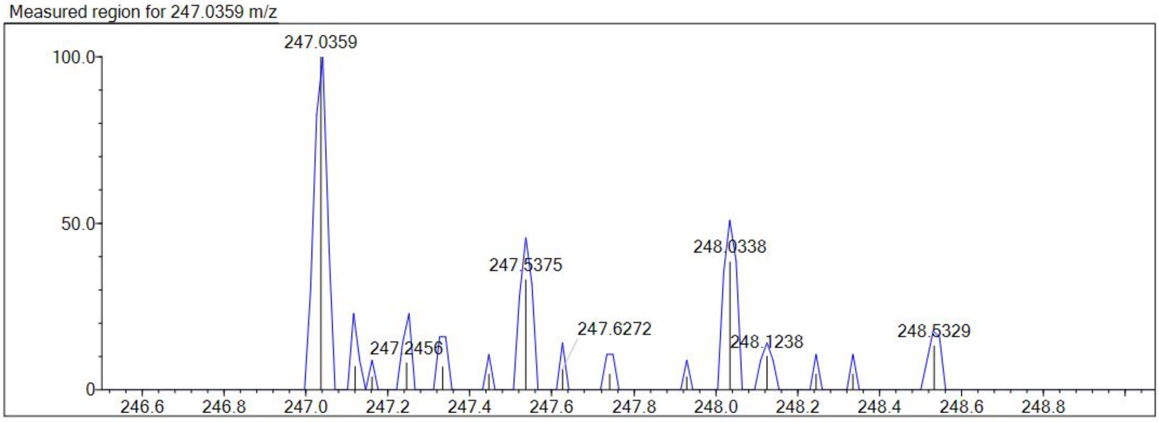

C23 H17 N6 O S2 Cl [M+2H]2+ : Predicted region for 247.0370 m/z

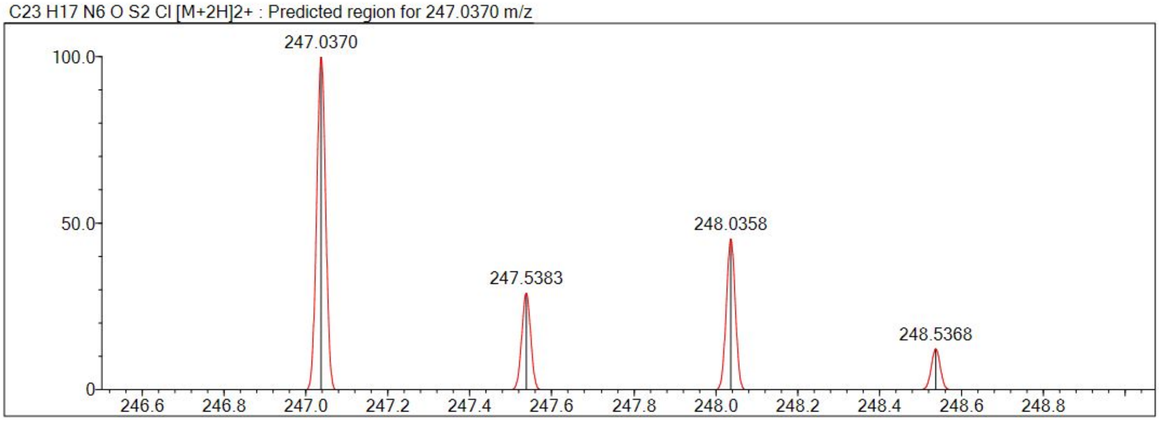

Rank	Score	Formula (M)	Ion	Meas. m/z	Pred. m/z	Df. (mDa)	Df. (ppm)	Iso	DBE
2	52.77	C23 H17 N6 O S2 Cl	[M+2H]2+	247.0359	247.0370	-1.1	-4.45	57.75	18.0

Figure S24. Mass spectrum of compound **8f**

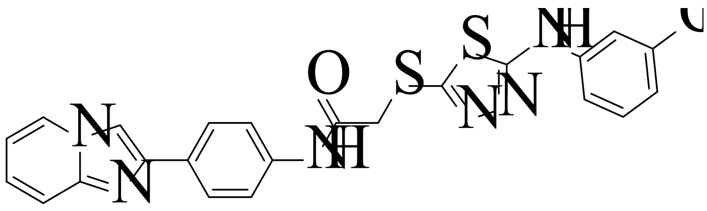

Figure S25. The chemical structure of compound **8g**

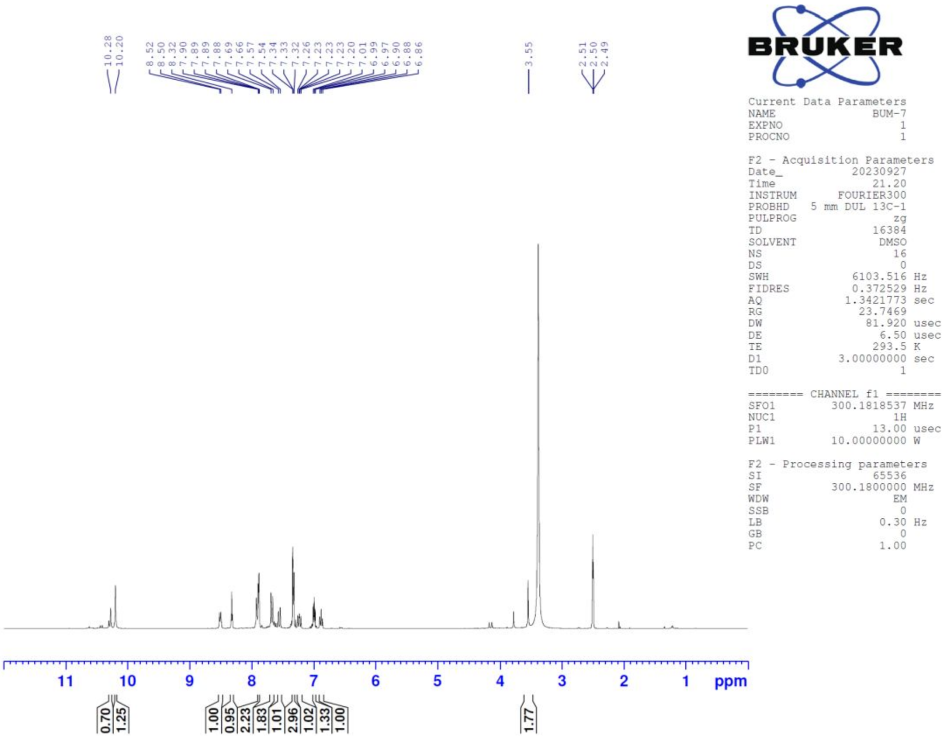

Figure S26. ^{1}H-NMR spectrum of compound **8g**

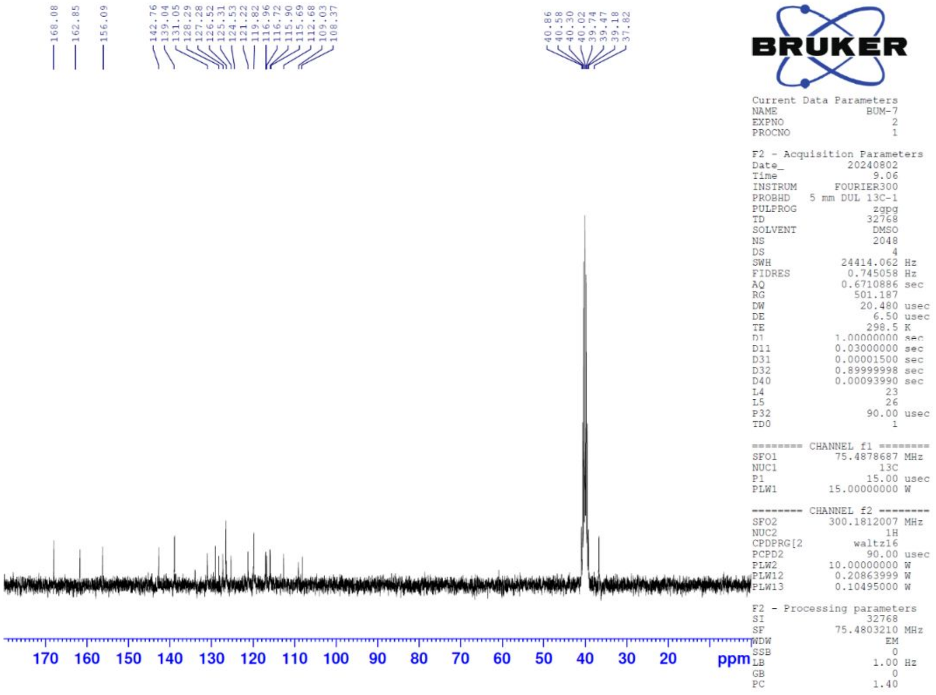

Figure S27. ^{13}C-NMR spectrum of compound **8g**

Data File: C:\LabSolutions\Data\Analiz\uac\BUM-7_230.lcd

Elmt	Val.	Min	Max	Elmt	Val.	Min	Max	Elmt	Val.	Min	Max	Elmt	Val.	Min	Max	Use Adduct
H	1	8	33	O	2	0	5	S	2	0	2	Ru	2	0	0	H
C	4	4	32	F	1	0	0	Cl	1	1	1	Pd	2	0	0	
N	3	6	6	P	3	0	0	Br	1	0	0	I	3	0	0	

Error Margin (ppm):	6	DBE Range:	0.0 - 30.0	Electron Ions:	both
HC Ratio:	unlimited	Apply N Rule:	no	Use MSn Info:	yes
Max Isotopes:	3	Isotope RI (%):	1.00	Isotope Res:	9000
MSn Iso RI (%):	10.00	MSn Logic Mode:	AND	Max Results:	50

Event#: 1 MS(E+) Ret. Time : 2.147 Scan# : 323

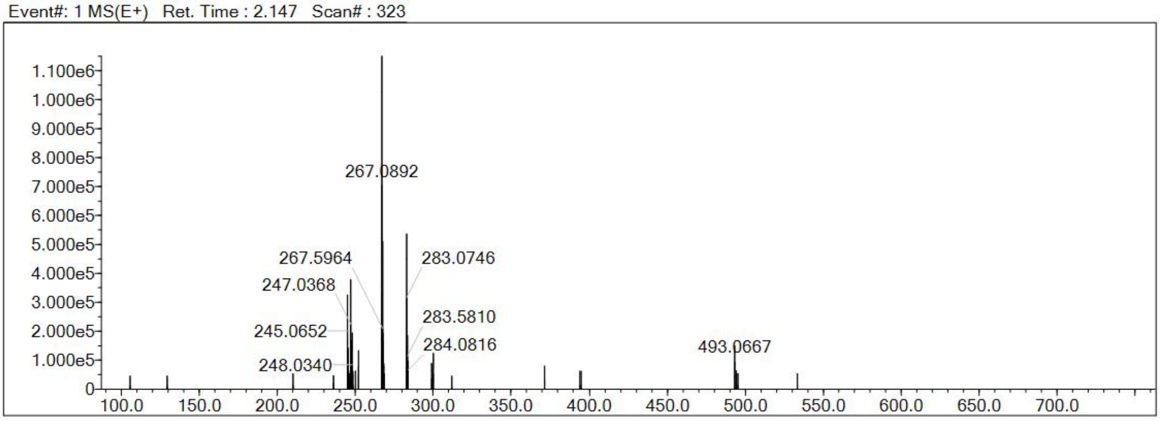

Measured region for 247.0368 m/z

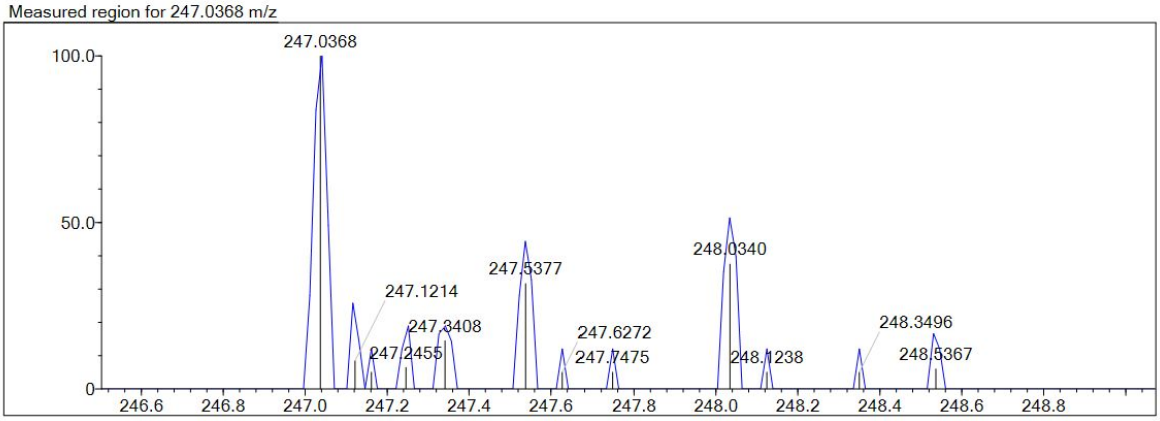

C23 H17 N6 O S2 Cl [M+2H]2+ : Predicted region for 247.0370 m/z

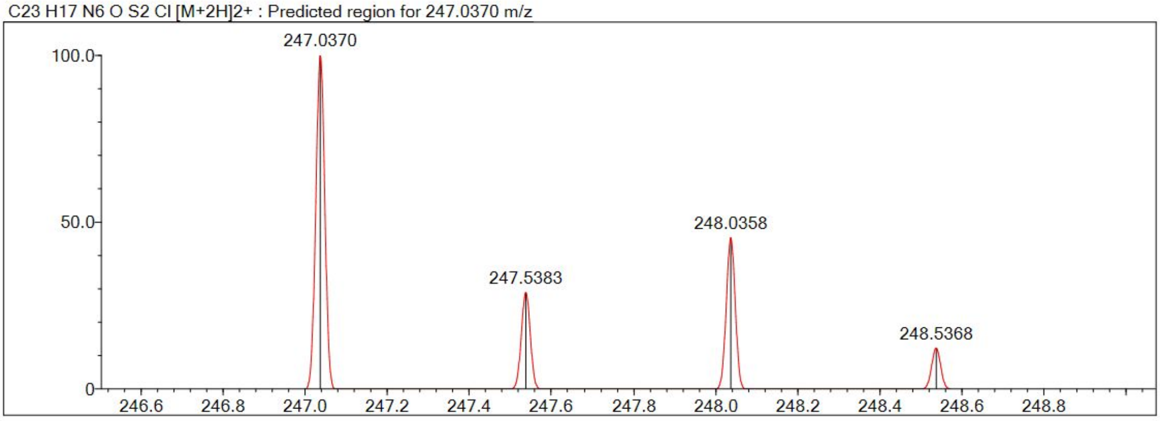

Rank	Score	Formula (M)	Ion	Meas. m/z	Pred. m/z	Df. (mDa)	Df. (ppm)	Iso	DBE
1	65.27	C23 H17 N6 O S2 Cl	[M+2H]2+	247.0368	247.0370	-0.2	-0.81	65.27	18.0

Figure S28. Mass spectrum of compound **8g**

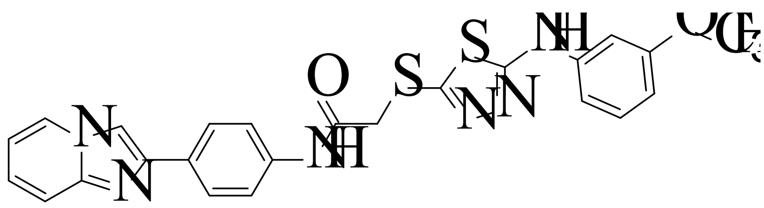

Figure S29. The chemical structure of compound **8h**

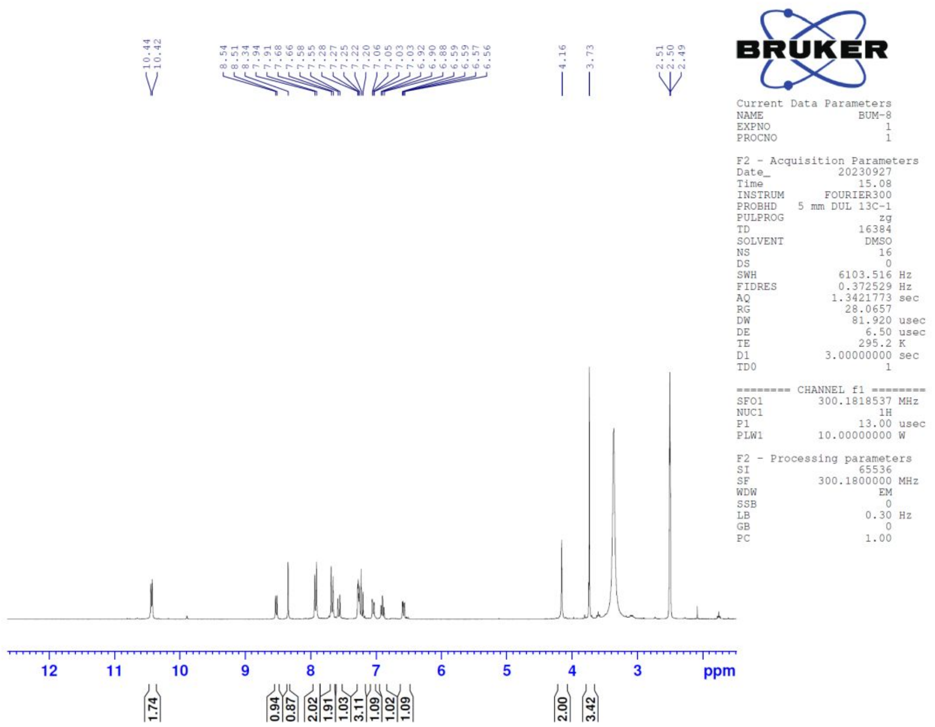

Figure S30. ^{1}H-NMR spectrum of compound **8h**

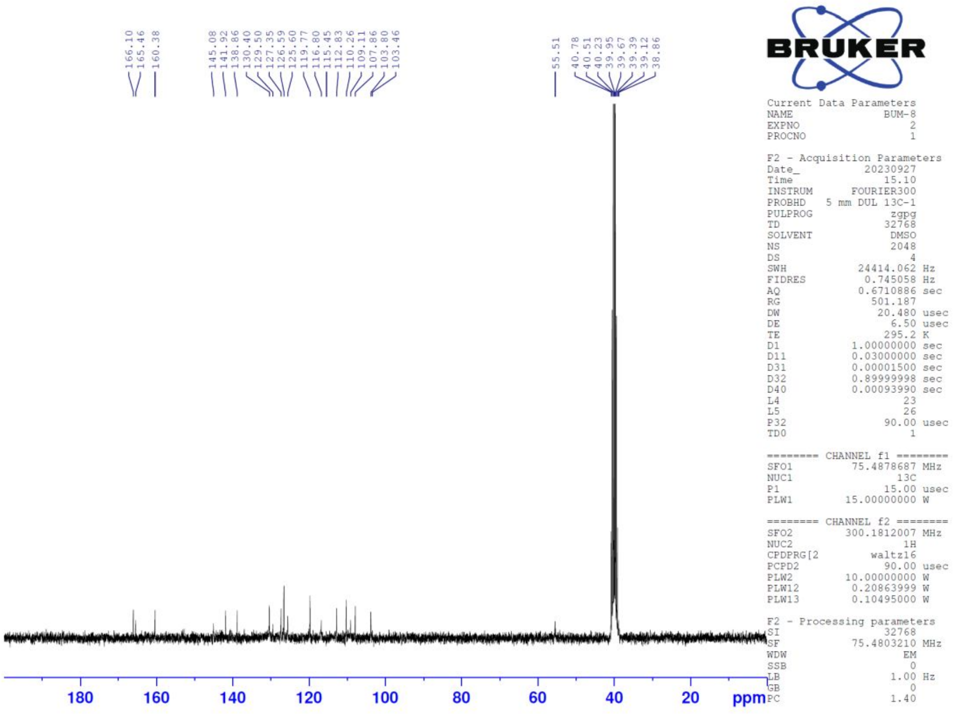

Figure S31. ^{13}C-NMR spectrum of compound **8h**

Formula Predictor Report - BUM-8_231.lcd

Page 1 of 1

Data File: C:\LabSolutions\Data\Analiz\uac\BUM-8_231.lcd

Elmt	Val.	Min	Max	Elmt	Val.	Min	Max	Elmt	Val.	Min	Max	Elmt	Val.	Min	Max	Use Adduct
H	1	8	33	O	2	0	5	S	2	0	2	Ru	2	0	0	H
C	4	4	32	F	1	0	0	Cl	1	0	0	Pd	2	0	0	
N	3	6	6	P	3	0	0	Br	1	0	0	I	3	0	0	

Error Margin (ppm):	6	DBE Range:	0.0 - 30.0	Electron Ions:	both
HC Ratio:	unlimited	Apply N Rule:	no	Use MSn Info:	yes
Max Isotopes:	3	Isotope RI (%):	1.00	Isotope Res:	9000
MSn Iso RI (%):	10.00	MSn Logic Mode:	AND	Max Results:	50

Event#: 1 MS(E+) Ret. Time : 2.067 Scan# : 311

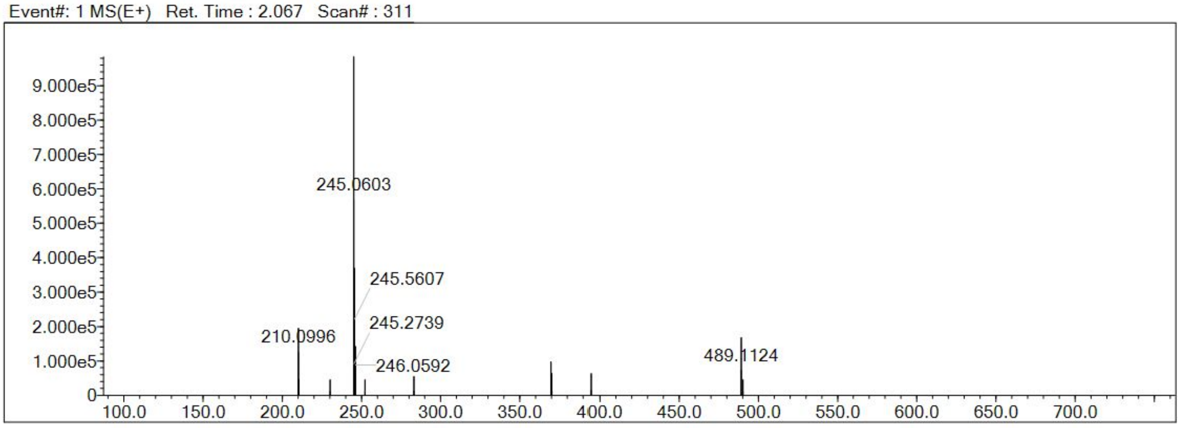

Measured region for 245.0603 m/z

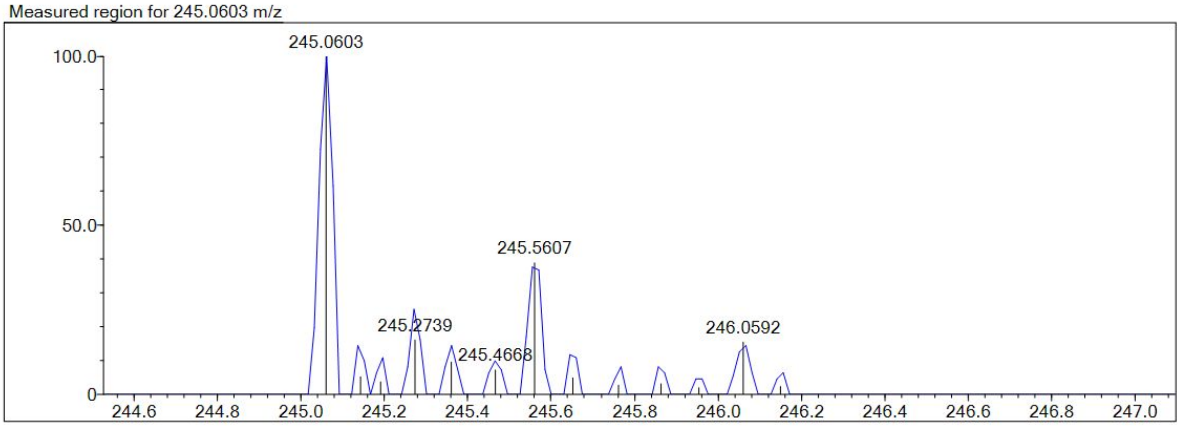

C24 H20 N6 O2 S2 [M+2H]2+ : Predicted region for 245.0617 m/z

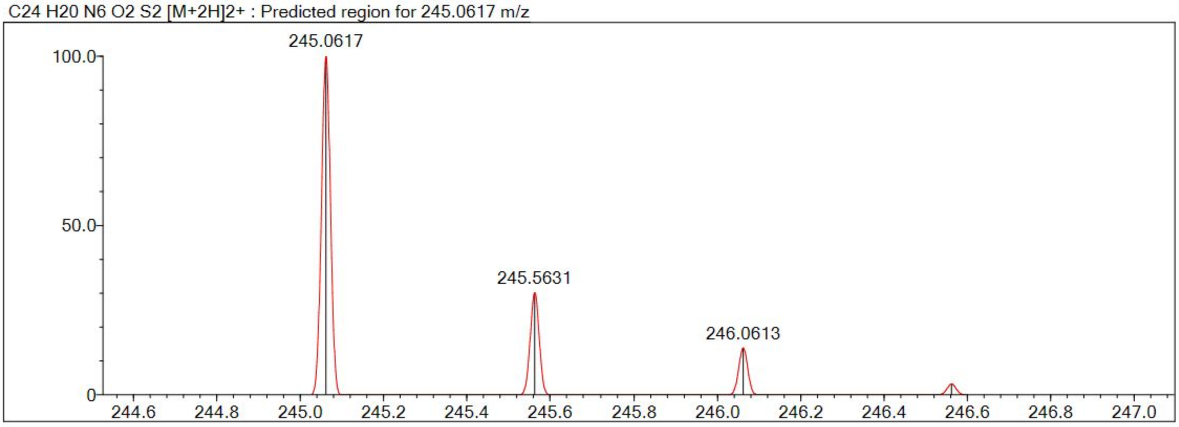

Rank	Score	Formula (M)	Ion	Meas. m/z	Pred. m/z	Df. (mDa)	Df. (ppm)	Iso	DBE
2	64.28	C24 H20 N6 O2 S2	[M+2H]2+	245.0603	245.0617	-1.4	-5.71	77.54	18.0

Figure S32. Mass spectrum of compound **8h**

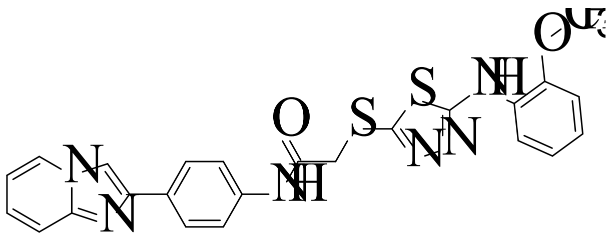

Figure S33. The chemical structure of compound **8i**

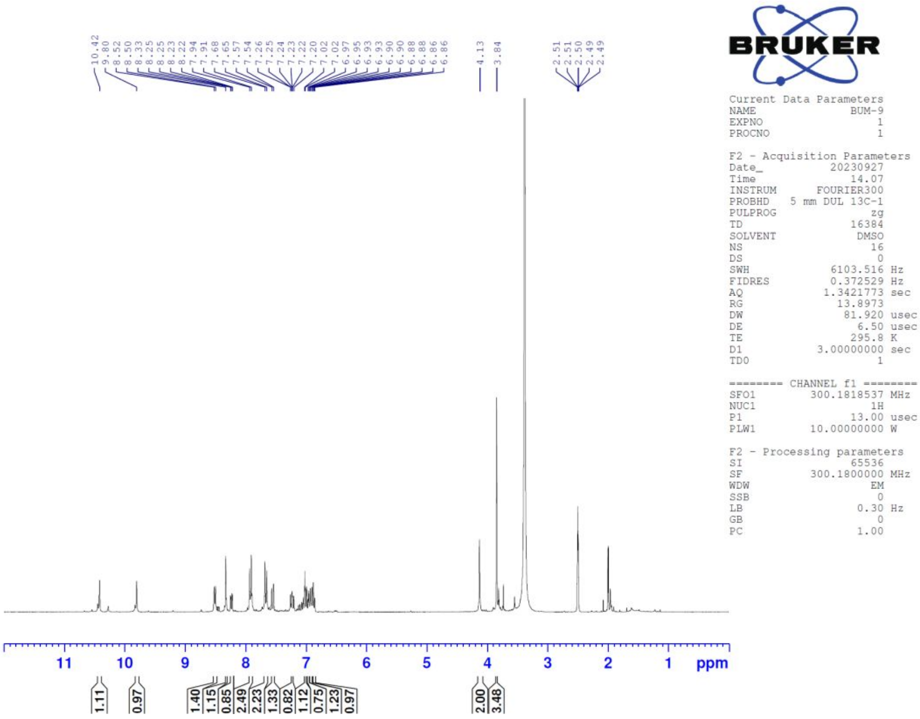

Figure S34. ^{1}H-NMR spectrum of compound **8i**

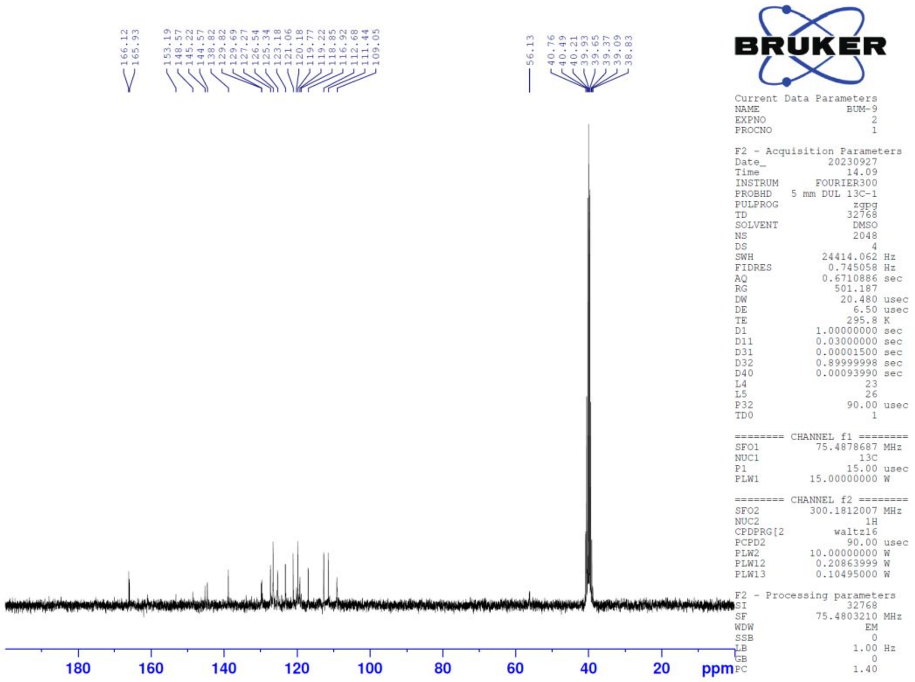

Figure S35. ^{13}C-NMR spectrum of compound **8i**

Data File: C:\LabSolutions\Data\Analiz\uac\BUM-9_2.lcd

Elmt	Val.	Min	Max	Elmt	Val.	Min	Max	Elmt	Val.	Min	Max	Elmt	Val.	Min	Max	Use Adduct
H	1	8	33	O	2	0	5	S	2	0	2	Ru	2	0	0	H
C	4	4	32	F	1	0	0	Cl	1	0	0	Pd	2	0	0	
N	3	6	6	P	3	0	0	Br	1	0	0	I	3	0	0	

Error Margin (ppm):	6	DBE Range:	0.0 - 30.0	Electron Ions:	both
HC Ratio:	unlimited	Apply N Rule:	no	Use MSn Info:	yes
Max Isotopes:	3	Isotope RI (%):	1.00	Isotope Res:	9000
MSn Iso RI (%):	10.00	MSn Logic Mode:	AND	Max Results:	50

Event#: 1 MS(E+) Ret. Time : 2.347 Scan# : 353

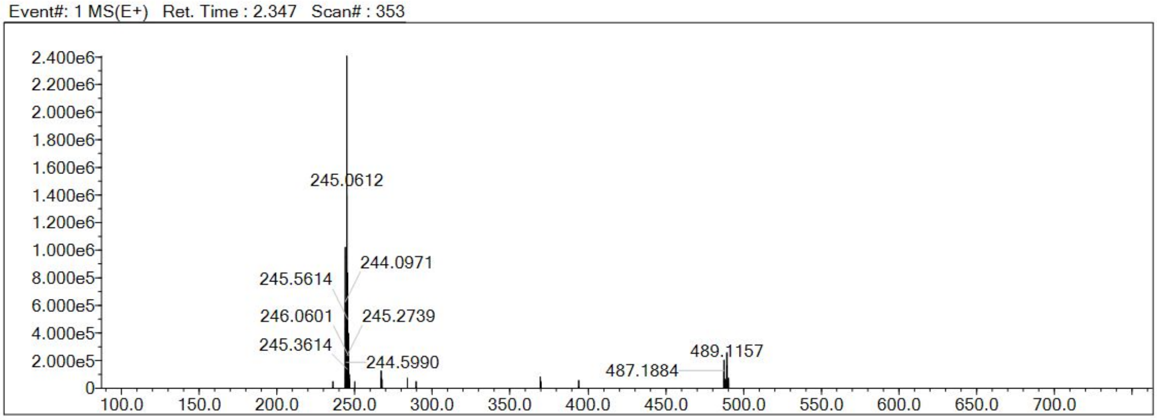

Measured region for 245.0612 m/z

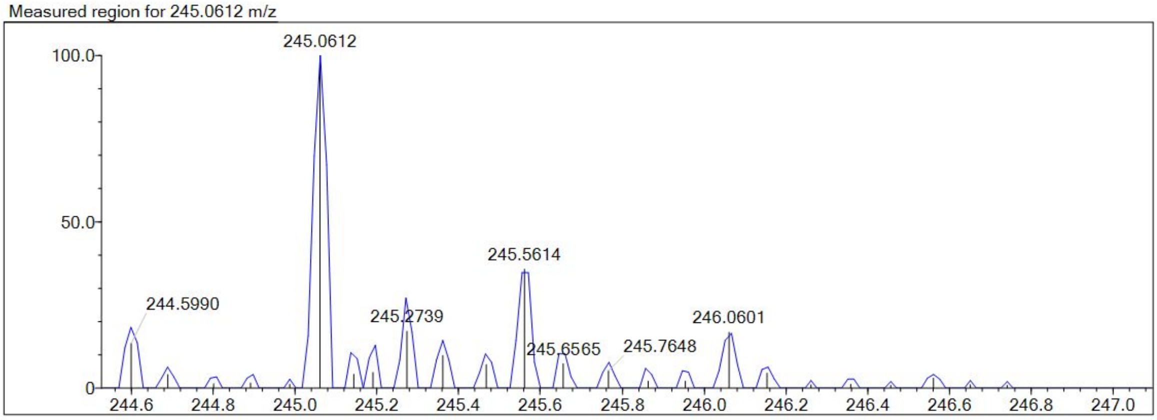

C24 H20 N6 O2 S2 [M+2H]2+ : Predicted region for 245.0617 m/z

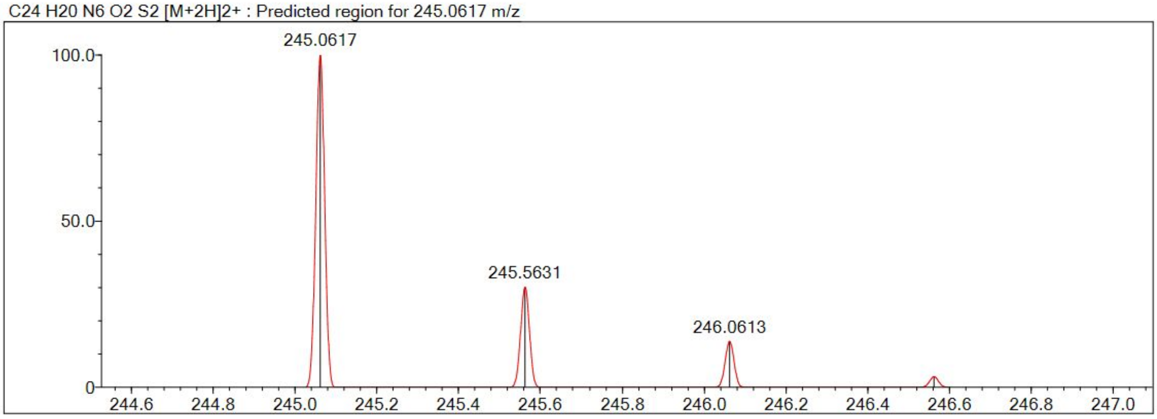

Rank	Score	Formula (M)	Ion	Meas. m/z	Pred. m/z	Df. (mDa)	Df. (ppm)	Iso	DBE
1	91.97	C24 H20 N6 O2 S2	[M+2H]2+	245.0612	245.0617	-0.5	-2.04	94.42	18.0

Figure S36. Mass spectrum of compound **8i**

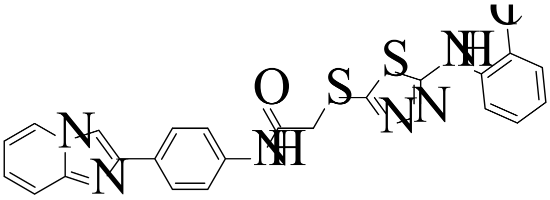

Figure S37. The chemical structure of compound **8j**

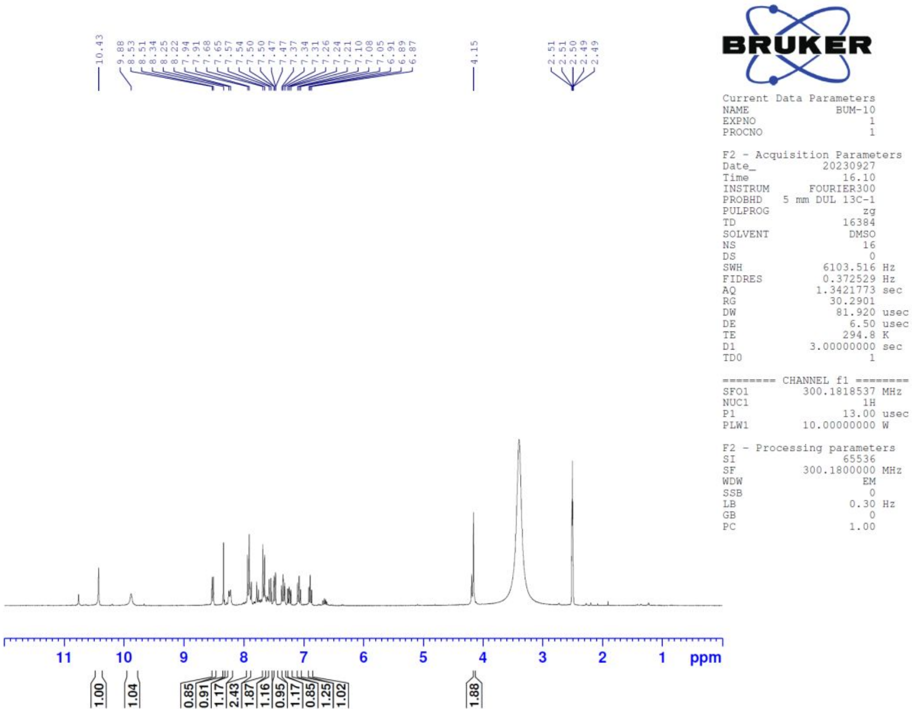

Figure S38. ^{1}H-NMR spectrum of compound **8j**

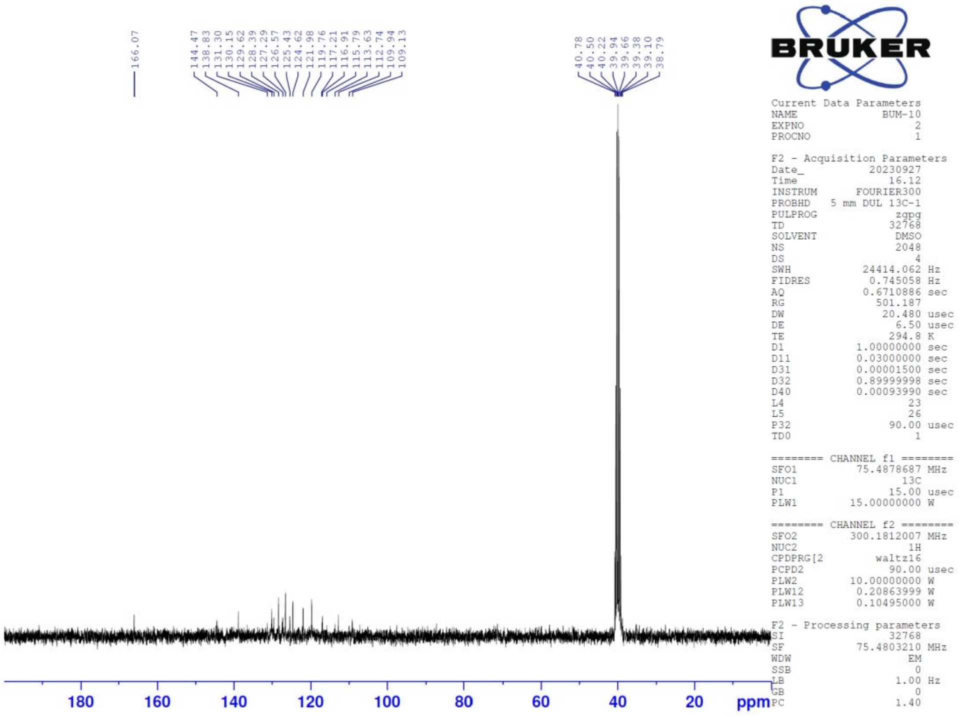

Figure S39. ^{13}C-NMR spectrum of compound **8j**

Data File: C:\LabSolutions\Data\Analiz\uac\BUM-10_3.lcd

Elmt	Val.	Min	Max	Elmt	Val.	Min	Max	Elmt	Val.	Min	Max	Elmt	Val.	Min	Max	Use Adduct
H	1	8	33	O	2	0	5	S	2	0	2	Ru	2	0	0	H
C	4	4	32	F	1	0	0	Cl	1	1	1	Pd	2	0	0	
N	3	6	6	P	3	0	0	Br	1	0	0	I	3	0	0	

Error Margin (ppm):	6	DBE Range:	0.0 - 30.0	Electron Ions:	both
HC Ratio:	unlimited	Apply N Rule:	no	Use MSn Info:	yes
Max Isotopes:	3	Isotope RI (%):	1.00	Isotope Res:	9000
MSn Iso RI (%):	10.00	MSn Logic Mode:	AND	Max Results:	50

Event#: 1 MS(E+) Ret. Time : 2.307 Scan# : 347

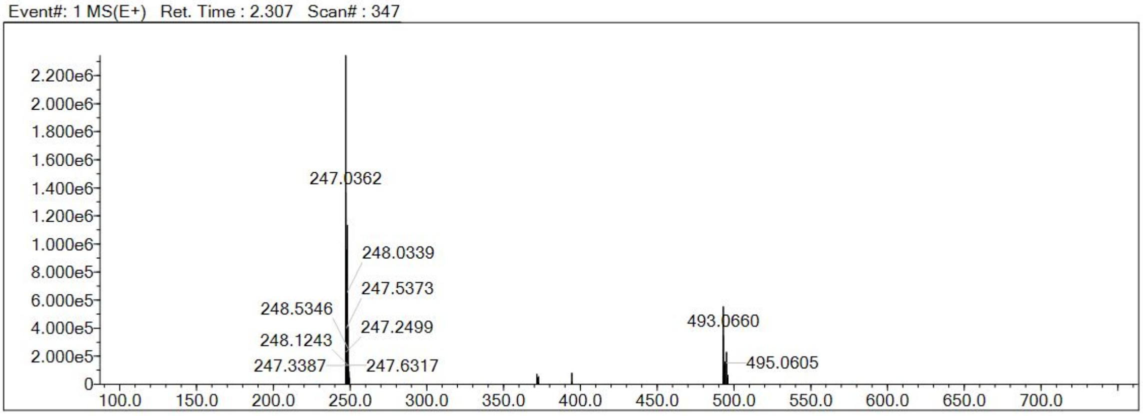

Measured region for 247.0362 m/z

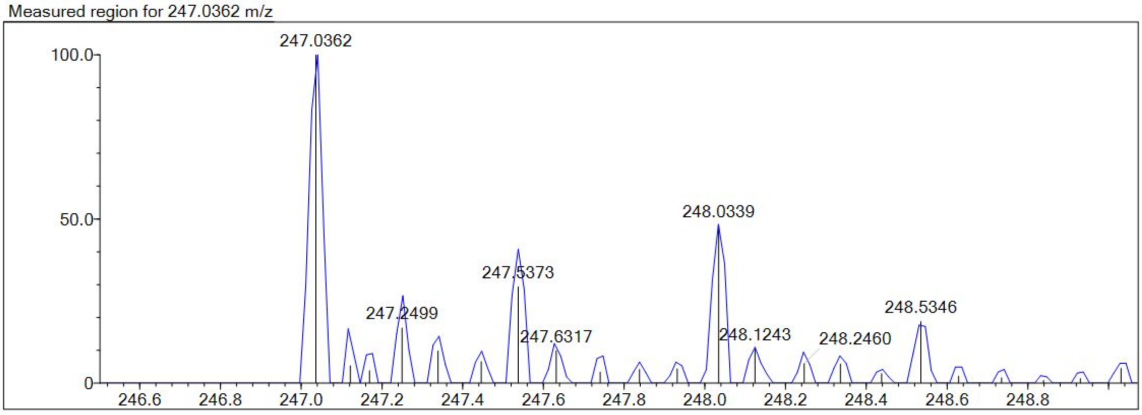

C23 H17 N6 O S2 Cl [M+2H]2+ : Predicted region for 247.0370 m/z

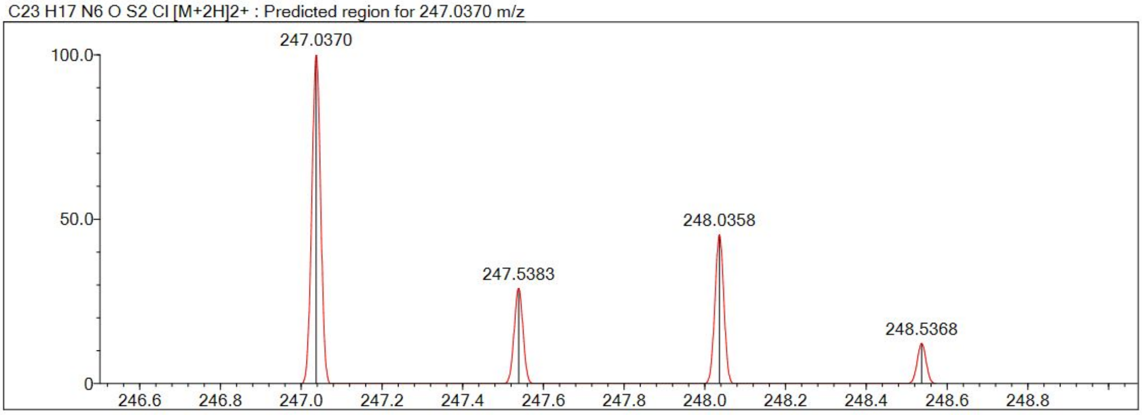

Rank	Score	Formula (M)	Ion	Meas. m/z	Pred. m/z	Df. (mDa)	Df. (ppm)	Iso	DBE
2	61.53	C23 H17 N6 O S2 Cl	[M+2H]2+	247.0362	247.0370	-0.8	-3.24	65.18	18.0

Figure S40. Mass spectrum of compound **8j**

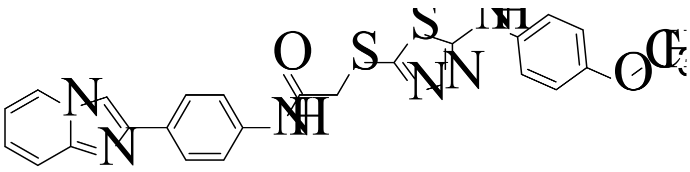

Figure S41. The chemical structure of compound **8k**

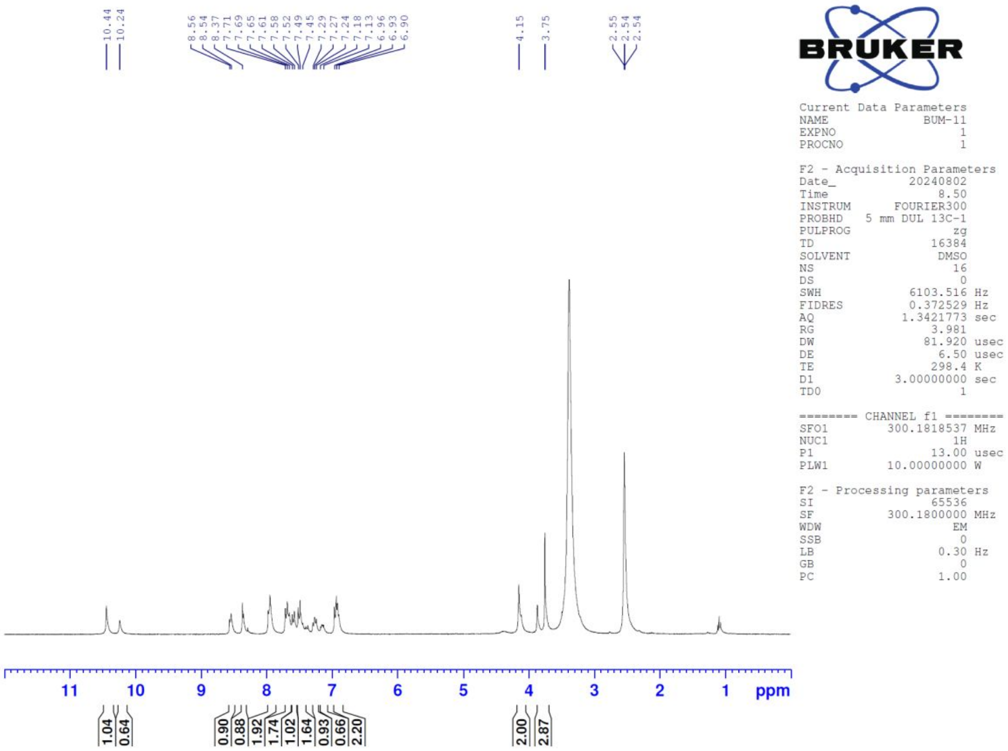

Figure S42. ^{1}H-NMR spectrum of compound **8k**

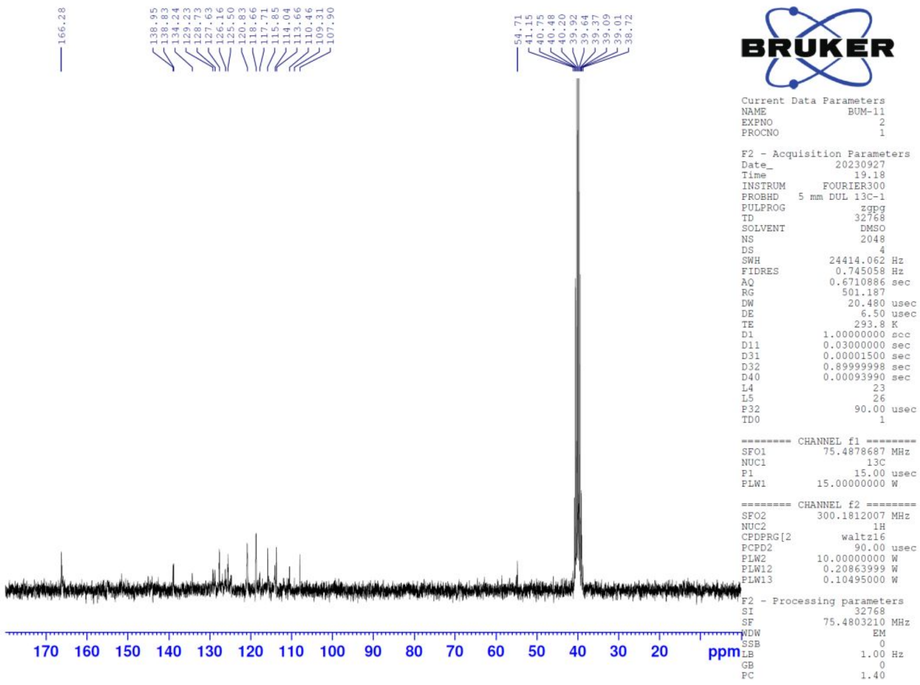

Figure S43. ^{13}C-NMR spectrum of compound **8k**

Formula Predictor Report - BUM-11_4.lcd Page 1 of 1

Data File: C:\LabSolutions\Data\Analiz\uac\BUM-11_4.lcd

Elmt	Val.	Min	Max	Elmt	Val.	Min	Max	Elmt	Val.	Min	Max	Elmt	Val.	Min	Max	Use Adduct
H	1	8	33	O	2	0	5	S	2	0	2	Ru	2	0	0	H
C	4	4	32	F	1	0	0	Cl	1	0	0	Pd	2	0	0	
N	3	6	6	P	3	0	0	Br	1	0	0	I	3	0	0	

Error Margin (ppm):	6	DBE Range:	0.0 - 30.0	Electron Ions:	both
HC Ratio:	unlimited	Apply N Rule:	no	Use MSn Info:	yes
Max Isotopes:	3	Isotope RI (%):	1.00	Isotope Res:	9000
MSn Iso RI (%):	10.00	MSn Logic Mode:	AND	Max Results:	50

Event#: 1 MS(E+) Ret. Time : 2.280 Scan# : 343

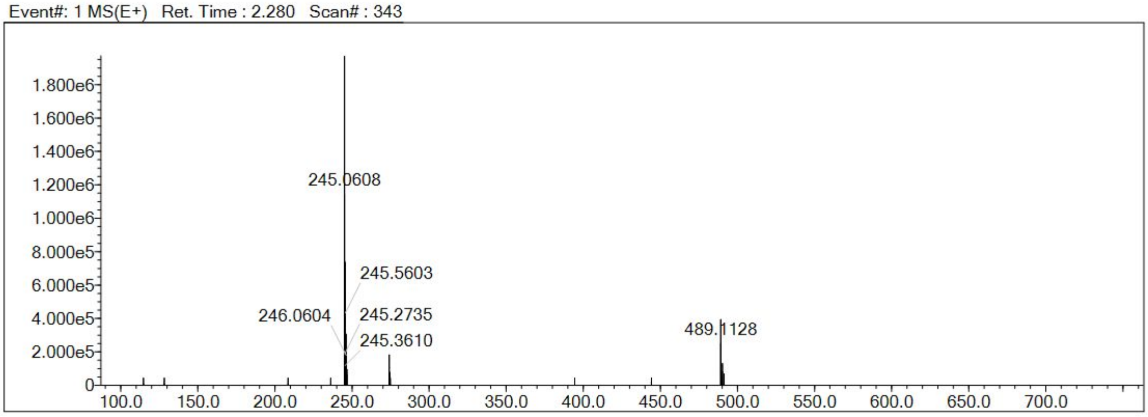

Measured region for 245.0608 m/z

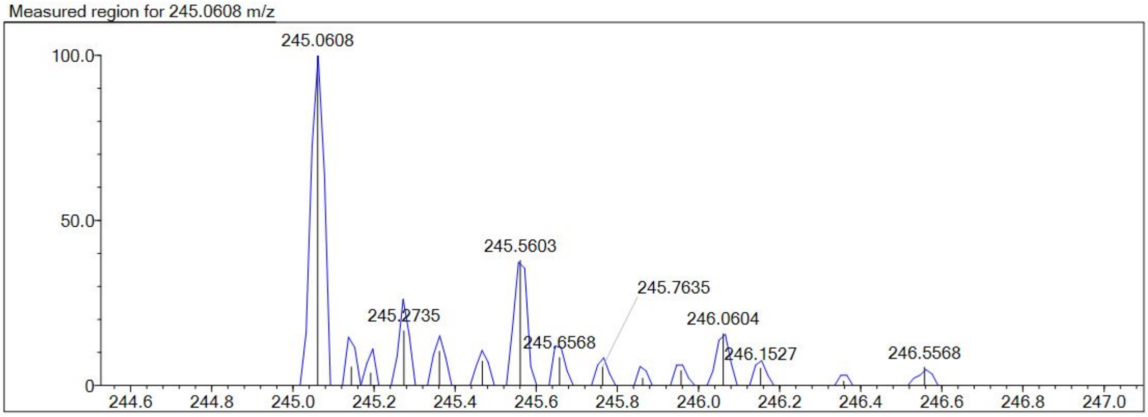

C24 H20 N6 O2 S2 [M+2H]2+ : Predicted region for 245.0617 m/z

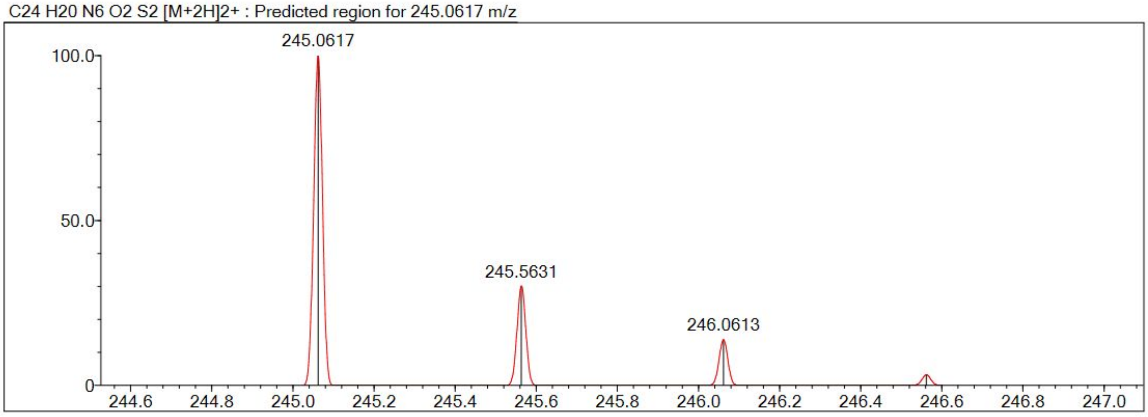

Rank	Score	Formula (M)	Ion	Meas. m/z	Pred. m/z	Df. (mDa)	Df. (ppm)	Iso	DBE
2	71.26	C24 H20 N6 O2 S2	[M+2H]2+	245.0608	245.0617	-0.9	-3.67	76.35	18.0

Figure S44. Mass spectrum of compound **8k**
